# Heterogeneity Driven Trapping at the Pore-Network Scale in Edwards Brown Dolomite

**DOI:** 10.1021/acs.energyfuels.5c04544

**Published:** 2025-12-16

**Authors:** Nihal Darraj, Sojwal Manoorkar, Catherine Spurin, Sajjad Foroughi, M. Saleh, Steffen Berg, Martin J. Blunt, Samuel Krevor

**Affiliations:** † Department of Earth Science and Engineering, 4615 Imperial College London, London SW7 2AZ, U.K.; ‡ Department of Geology, 26656 Ghent University, Krijgslaan 281, Ghent 9000, Belgium; § Energy Science and Engineering, 6429 Stanford University, Palo Alto, California 94305, United States; ∥ Shell Global Solutions International B.V., Grasweg 31, Amsterdam 1031 WG, Netherlands

## Abstract

Trapping is a key control governing the stability and long-term containment of CO_2_ within geological storage formations, with residual trapping at the pore scale being well established and routinely incorporated into reservoir simulation models. In contrast, integrating the effects of capillary trapping arising from spatial variability in capillary entry pressure at the micron to centimeter scale remains a challenge for field-scale models, despite clear evidence of its influence on plume migration. Studying pore-scale heterogeneity allows direct quantification of how heterogeneity in pore connectivity and throat geometry translates into capillary entry pressures and snap-off mechanisms, which ultimately control trapping efficiency and is not often resolved at the continuum scale. In this study, we performed flow experiments with brine and decane under capillary-dominated conditions (*C*_a_ = 2.6 × 10^–7^) while acquiring time-resolved 3D micro-CT images at 5.6 μm voxel size on a 12 mm by 60 mm rock sample. Fractional-flow drainage and imbibition steps were imaged at steady state. Segmented volumes were analyzed with pore-network analysis and trapped volumes were investigated with ganglia volume and count analysis. The sample contains a downstream low-porosity region that acts as a partial capillary barrier. This region remained brine-saturated even during 100% decane injection, indicating entry pressures above the applied capillary driving force. Pore-network analysis showed limited connectivity where the resolved coordination number is approximately only 2, with more than 30% of pores connected by two or fewer throats. The relationship between local initial and residual saturations shows that, within the barrier region, the two values are nearly equivalent, indicating negligible displacement of the mobile phase demonstrating minimal displacement and enhanced trapping. The ganglia analysis shows that the volume and count of ganglia trapped behind the barrier remained elevated after imbibition. These results show that capillary barriers increase immobilization while reducing accessible pore volume. This has an influence on plume migration pathways and should be captured in upscaled models for storage in heterogeneous formations.

## Graphical abstract

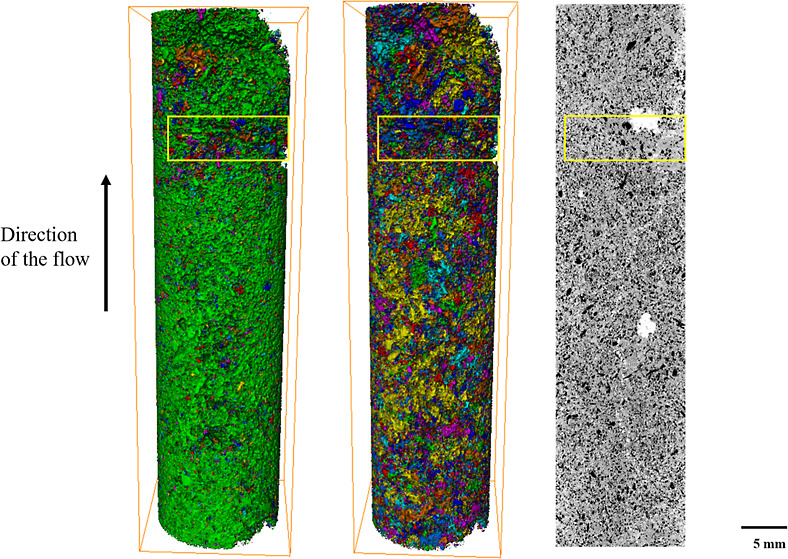

## 1 Introduction

One of the main requirements to ensure secure, and environmentally responsible CO_2_ storage is to understand subsurface migration, where multiphase flow is controlled by buoyancy, capillary pressure, and viscous forces, together with geologic heterogeneity and operational parameters.^1,2^ The impact of small-scale heterogeneities, capillary heterogeneity over centimeter length scales in particular, has a significant influence on field-scale CO_2_ migration.^3–12^

Furthermore, capillary heterogeneity is a critical factor influencing CO_2_ residual trapping.^7,13^ Residual trapping refers to the trapping of CO_2_ in pore spaces by capillary forces once injection stops and brine re-enters the formation. Trapping can be quantified by fitting empirical relationships to the measured initial-residual saturation relationship.^14^ This process leaves behind disconnected ganglia of CO_2_, where trapping is largely limited to pore-scale features.^15^ However, Krevor et al.^13^ demonstrated that in heterogeneous formations, small-scale variations in capillary entry pressure can lead to the immobilization of larger segments of the CO_2_ plume. These local capillary barriers enhance trapping beyond the pore scale, indicating that residual trapping can comprise of both pore-scale immobilization and heterogeneity-driven capillary entrapment. This dual mechanism significantly increases the overall volume of CO_2_ that can be retained in the subsurface.

Carbonate rock samples present an important case study, as they host approximately 60% of the world’s oil reserves making them prime candidates for CO_2_ storage.^16^ Moreover, compared to sandstones, carbonate rocks exhibit a diverse array of pore and throat types and sizes. This inherent complexity leads to unique fluid flow and trapping behavior.^17–19^ Some carbonate formations have been reported to contain significant microporosity, contributing as much as 10–60% of the total porosity.^20,21^ In such heterogeneous carbonate rocks, the non-wetting phase may preferentially invade the more accessible macropores, leaving large volumes of brine-saturated microporosity untouched or leading to the isolation of CO_2_ in disconnected pore regions and higher trapping efficiency.^9,22^

In this work, we present a pore-scale experimental investigation of multiphase flow in Edwards Brown rock sample to elucidate how small-scale heterogeneity influences CO_2_ trapping and saturation profiles. The rationale for selecting Edwards Brown dolomite lies in its highly complex pore structure, which makes it an effective analogue for heterogeneous carbonate reservoirs. The rock exhibits multiscale porosity, combining microporous dolomite crystals with larger vugs and moldic pores, which together produce a broad distribution of pore throat sizes and capillary entry pressures. Unlike many layered carbonates and sandstones, Edwards Brown displays spatially isotropic heterogeneity, where low-permeability patches and vug-rich zones are distributed irregularly throughout the matrix rather than forming distinct stratification.^18,23^ This heterogeneous arrangement amplifies capillary contrasts and enhances the likelihood of local barriers and snap-off, providing an ideal system for investigating pore-scale trapping mechanisms. The sample size (12 mm in diameter and 60 mm in length) is large enough to capture continuum-scale properties, while still being small enough to resolve pore-scale phenomena. This includes direct observation of fluid–fluid interfaces, connectivity of the pore network, and the displacement mechanisms that control trapping.

We utilize X-ray imaging with a 5.6 μm voxel size combined with core-flooding experiments to directly observe fluid displacement patterns under capillary-dominated flow conditions. By quantitatively linking spatial variations in pore structure to local fluid occupancy and residual non-wetting phase trapping, we bridge the gap between pore-scale processes and continuum-scale implications. The findings provide insight into how heterogeneity in carbonates can lead to different trapping efficiencies within the same sample. These observations apply to Edwards Brown carbonate and similar rocks exhibiting the same scale and nature of heterogeneity.

## 2 Materials and Methods

### 2.1 Rock Sample and Fluids

The rock used in this study is Edwards Brown dolomite, quarried from an Upper Cretaceous carbonate formation in Texas, USA. It is a dolomitised limestone with pronounced diagenetic heterogeneity. Mineralogical analysis indicates that dolomite is the dominant phase, with minor calcite and trace quartz impurities.^24^

Measured on a cylindrical sample that is 3.8 cm in diameter and 15 cm long the total porosity of the Edwards Brown dolomite sample was measured at approximately 26% using a medical CT scanner (using dry scan-wet scan correlation).^18^ Reported mercury injection capillary pressure results for Edwards Brown indicate a pore-throat radius range of approximately 0.1 to 20 μm with a mean of 10.5 μm.^25^

We analyzed a cylindrical rock plug (12 mm in diameter and 60 mm in length). The plug’s porosity is ∼22% as determined by 5.6 μm voxel size X-ray imaging considering only resolvable pores (Figure 1), and its permeability to brine at ambient temperature and pressure was measured to be approximately 110 ± 10 mD.

**Figure 1:**
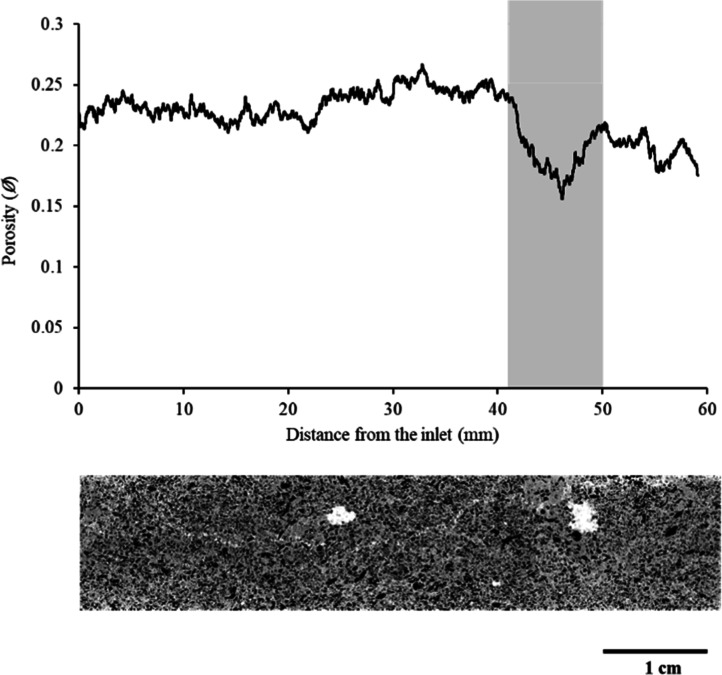
Top panel is a one-dimensional porosity profile along the sample’s length (averaged over cross-sectional slices) indicating the low porosity region with the shading. The bottom image shows the middle slice of a representative two-dimensional cross-section of a 3D micro-CT image through a region of interest (gray is the rock matrix, black is the pore space, and white is mineral).

The sample was oven-dried under vacuum at 80 °C for 72 h prior to the experiment. Two fluid phases were used in the flooding experiments: an aqueous brine as the wetting phase and an oil (*n*-decane) as the non-wetting phase. The brine was a KI (potassium iodide) solution (30 wt % KI, ∼2.58 mol/kg), chosen to provide X-ray contrast with the non-wetting phase.^26^ The density of the brine was measured to be 1263 ± 2 kg/m^3^ and the viscosity was 0.822 ± 0.013 mPa s at experimental conditions,^27^ the salinity of the KI brine is 2.4 molar, which is greater than that of seawater at 0.6 molar, but in the same range of highly saline carbonate reservoirs where brine molarity can range from 2 to 3 molar.^58–60^ Decane (*n*-decane, C_10_H_22_) was selected as an analogue for CO_2_: *n*-decane is frequently used as a proxy for supercritical CO_2_ due to its similar capillary displacement characteristics and convenient experimental handling.^28,29^ The decane–brine interfacial tension (IFT) was 47 ± 2 mN/m,^30^ is on the same order of magnitude as supercritical CO_2_–brine systems, which is typically 20–35 mN/m at reservoir conditions.^31^ Moreover, as long as the capillary number remains well below 10^–5^, the system is in the capillary-dominated regime, and macroscopic properties such as relative permeability and residual non-wetting phase saturation are insensitive to the absolute value of interfacial tension.^32,33^ Around 1000 m depth, supercritical CO_2_ begins to mimic two key properties of a light hydrocarbon (like decane): a density on the order of 600–700 kg/m^3^ and interfacial tension ∼47 mN/m. By 1500–2000 m, CO_2_’s density essentially equals that of decane. However, CO_2_’s viscosity remains an order-of-magnitude lower than decane’s at all depths.^30^

Furthermore, decane remains liquid at atmospheric conditions and is chemically inert with brine, enabling straightforward laboratory handling and X-ray imaging without specialized high-pressure equipment.^34^ Using decane allows a controlled, capillary-dominated multiphase displacement experiments under room temperature conditions, while still capturing physics relevant to CO_2_ injection in brine-filled rock. The *n*-decane/brine system is employed here as a controlled analogue to explore capillary-dominated flow behavior. While it does not replicate the exact physical properties of CO_2_, the flow remains within the same dimensionless regime where displacement mechanisms are governed by pore geometry and wettability rather than absolute fluid properties.

### 2.2 Experimental Apparatus and Imaging Procedure

The sample holder was of a (PEEK) material, with the rock wrapped in a flexible fluoropolymer (Viton) sleeve. This assembly was placed inside a Zeiss Xradia 510 Versa X-ray micro-CT scanner for in situ imaging.

Figure 3 provides a schematic of the apparatus. The flow system was configured with six high-precision Teledyne ISCO pumps, enabling the control over fluid phase injection, application of confining pressure, and regulation of system pressure through a dedicated back pressure pump. All core-flooding and imaging experiments were conducted at room temperature (22 ± 1 °C) and atmospheric pressure (1 bar; 0.1 MPa). However, the Edwards Brown Dolomite represents a shallow to mid-depth carbonate reservoir, where typical in situ conditions are on the order of 50–80 °C and 10–15 MPa. These values are provided for reference only, as the present experiments were designed to examine capillary-dominated displacement mechanisms under controlled laboratory conditions. During imaging, a constant confining pressure of 3.5 MPa (applied with deionized water around the Viton sleeve) was maintained to emulate an in situ overburden stress and to prevent any bypass flow along the sample walls. The pore pressure inside the sample was maintained at 0.3 MPa at the outlet (backpressure). Micro-CT scans were acquired at various stages of the flow experiment to visualize the internal distribution of fluids. Each scan was performed at an X-ray energy of 75 kV and power of 6.5 W. A large field-of-view detector was used to capture the entire sample cross-section at a voxel size of 5.6 μm. For each static scan, 3200 projection images were collected over 360° rotation, which, after reconstruction (using Zeiss Reconstruction software with appropriate center-shift and beam-hardening corrections), yielded a three-dimensional image of the X-ray attenuation within the sample.

**Figure 2:**
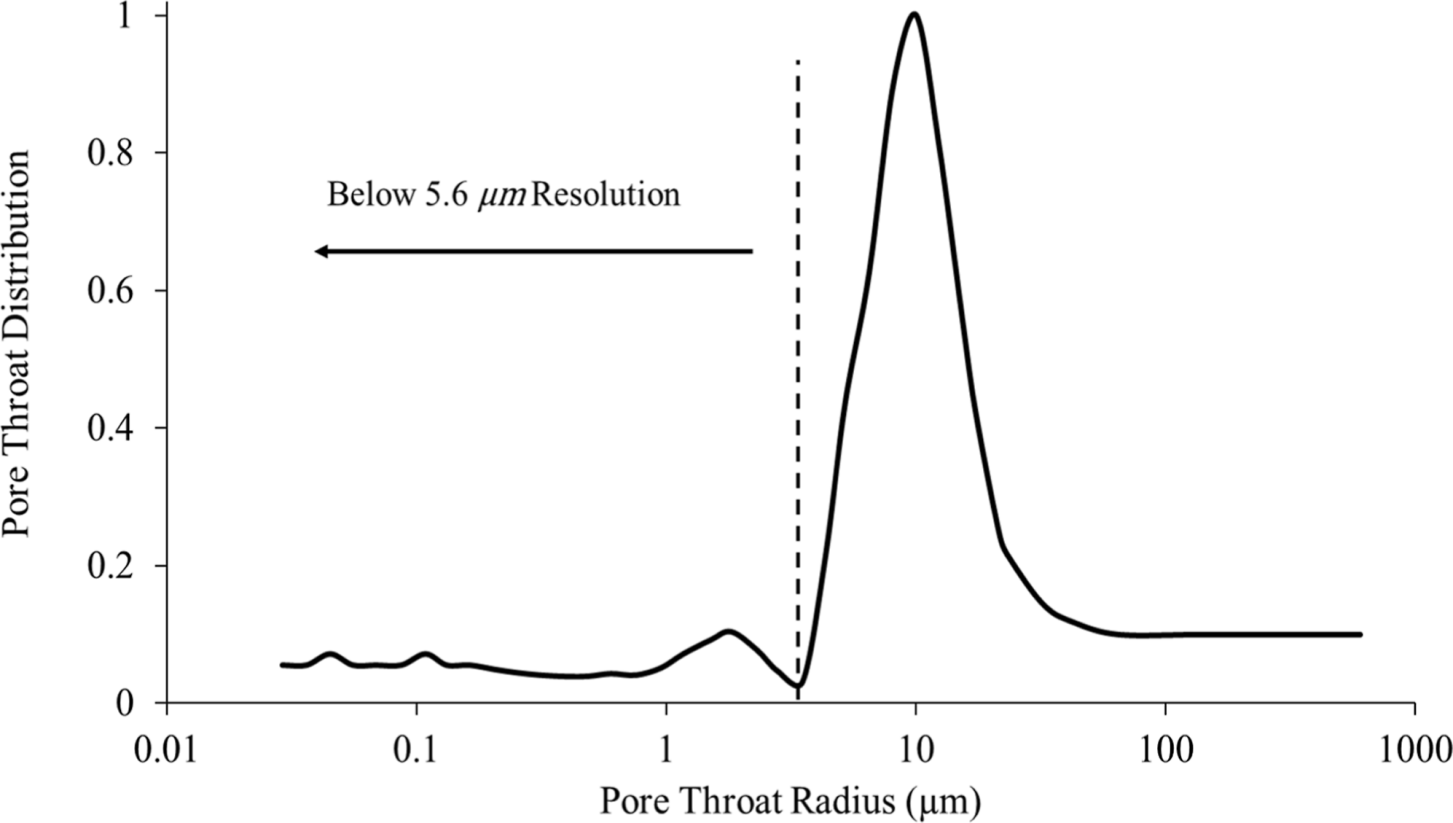
Mercury intrusion capillary pressure data that has been converted to show the distribution of pore-throat radius, normalized such that the maximum value is 1. Throats of radius below the voxel size of the image (5.6 μm) cannot be resolved.

**Figure 3:**
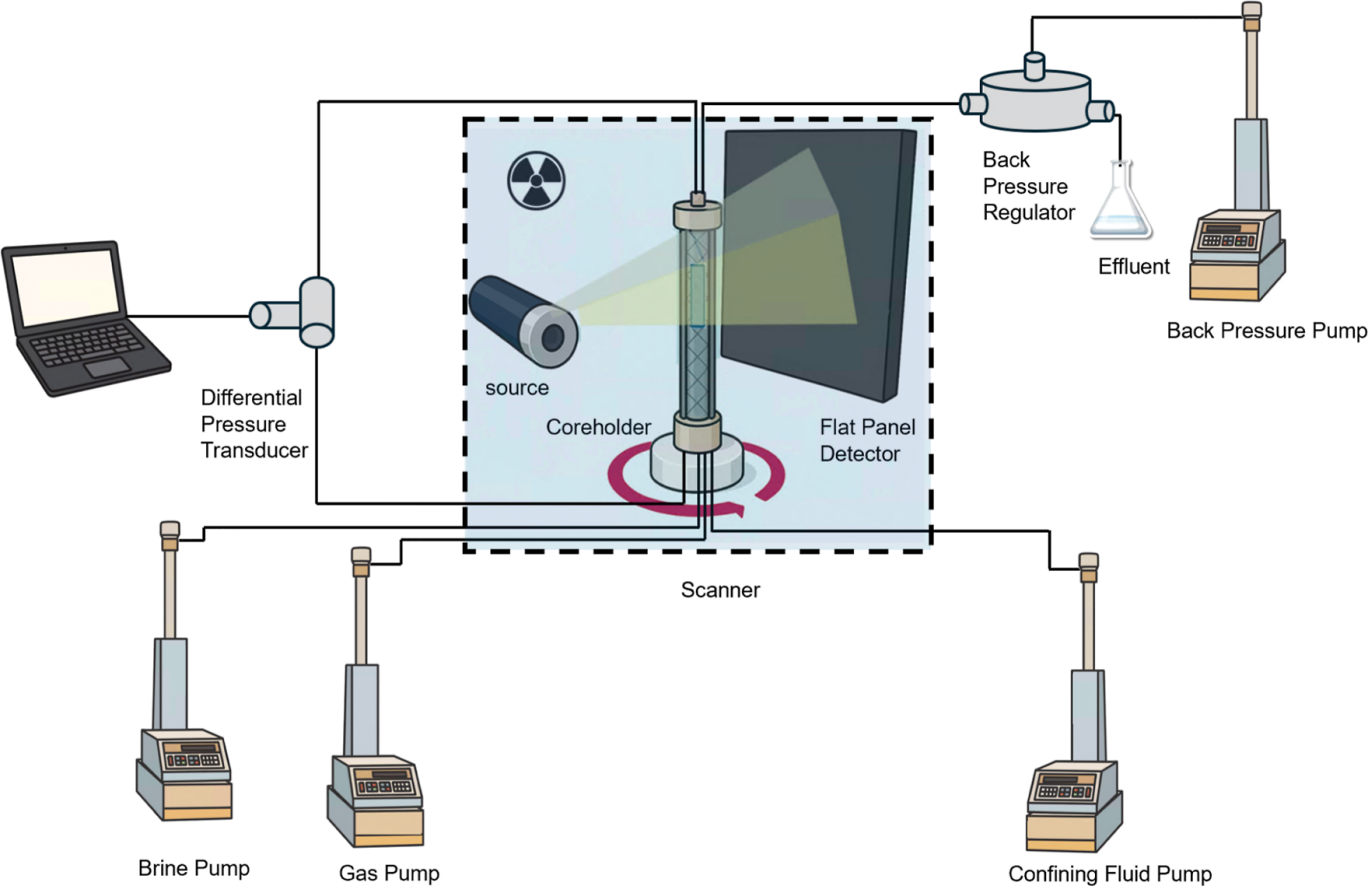
Experimental apparatus.

The resolution (∼5.6 μm) is sufficient to resolve the macropores and larger pore throats in the dolomite, though the finest microporosity remains below this detection limit (Figure 2).

The reconstructed gray scale images distinguish solid mineral matrix (high attenuation), brine (moderate attenuation due to KI content), and decane (low attenuation, similar to air) by their X-ray absorption contrast (Figure 4b). The multiphase flow experiment consisted of a sequence of steps designed to initially saturate the sample with CO_2_ to purge the air, then establish a brine-saturated condition, and finally perform controlled drainage and imbibition cycles while imaging at steady-state conditions.

**Figure 4:**
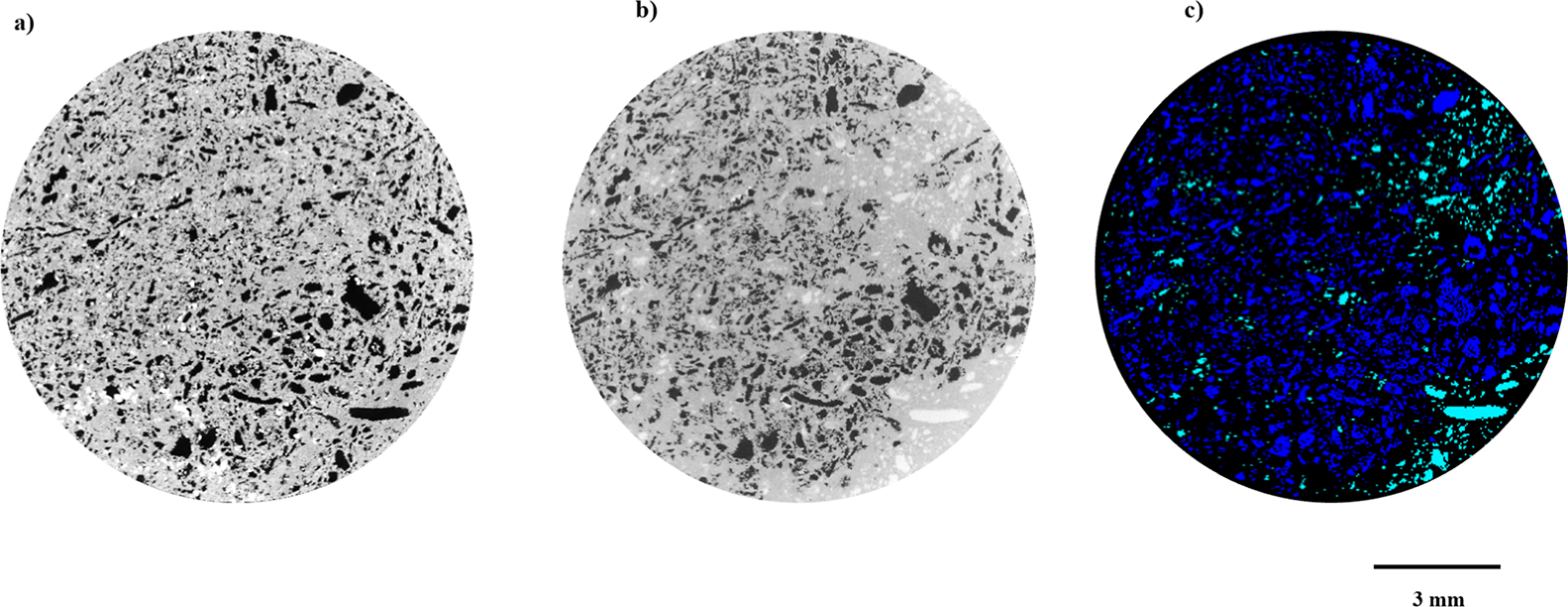
Two-dimensional cross sections of a 3D micro-CT images at the end of drainage (*f*_w_ = 0): (a) dry scan, rock in gray and pores in black; (b) filtered flow scan, showing rock in gray, decane in black, and brine in white; (c) segmented image, with rock represented in black, decane in blue, and brine in green.

The overall procedure was as follows:

(1) Initialisation: The dry sample was assembled in the coreholder and placed inside the scanner. A net confining pressure of 3.2 MPa was applied around the sleeve and left for 3 h with close monitoring of the confining pump. This step ensured the sample was isolated from the confining fluid and there is no flow bypass along the sleeve–rock interface once fluids were introduced. The dry scan was taken.
(2) CO_2_ Saturation: Gaseous CO_2_ was injected into the sample at a pore pressure of ∼1 MPa for 30 min displacing air, which here acts as an initial pore-filling gas.
(3) Brine Saturation: Brine was injected into the sample at a constant flow rate of 1 mL/min displacing the CO_2_ gas. This brine flooding continued until the sample was fully saturated with brine. During this and all other subsequent stages, the pressure difference across the sample was measured. During this step, the absolute permeability of the rock was measured by varying the flow rate and recording the pressure differential, then using Darcy’s law and pressure correction. The brine filled scan was taken.
(4) Drainage (CO_2_ analogue injection): After establishing a brine-saturated state, we initiated a drainage sequence. Using the dual pump system, decane and brine were coinjected at a fixed total flow rate of 0.1 mL/min, while gradually increasing the fractional flow of decane. We conducted a series of steady-state fractional flow steps with brine fractional flows of *f*_w_ = 0.9, 0.75, 0.5, 0.25, 0.05, 0 (100% decane). Each step was maintained until pressure equilibrium was reached, indicating steady state, and then a 3D micro-CT scan was taken. The capillary number during these flows was (*C*_a_ = μ·*q*/σ ≈ 2.6 × 10^–7^, using the decane viscosity and brine–decane IFT), ensuring that the displacement was capillary-dominated and representative of immiscible flow regimes in the subsurface. By the end of the drainage cycle (100% decane injection), the sample’s pore space had a high saturation of decane, with brine remaining only in the most difficult-to-invade pores.
(5) Imbibition (brine reinjection): Next, an imbibition sequence was performed to simulate the postinjection brine encroachment and investigate trapping. We gradually increased the brine fraction in coinjection steps reversing the sequence: brine fractional flows *f*_w_ = 0.05, 0.25, 0.5, 0.75, 0.9, and finally 1.0 (for 100% brine). Again, each fractional flow condition was held until steady-state was achieved before imaging. After the final imbibition step (100% brine), the sample was left with a certain amount of residual decane trapped in the pore space, analogous to residually trapped CO_2_ after a water flush or plume migration. Micro-CT scans were taken at the end of each fractional flow step in the imbibition cycle.

At each imaged steady state (both during drainage and imbibition), the three-dimensional distribution of brine and decane inside the pore space was mapped from the micro-CT images. This stepwise variation in fractional flow was designed to probe the effect of pore-scale heterogeneity on saturation evolution under capillary-dominated conditions. Since the displacement mechanisms are path-dependent in such regimes, each steady-state image captures the cumulative impact of injection history on non-wetting phase configuration. To reach the residual and irreducible saturations, injection was carried out for about 6 h (approximately 24 pore volumes) prior to scanning to ensure steady-state conditions are reached. Image processing steps (described below) were applied to segment the gray scale images into binary phases (brine and decane) and to quantify local saturations, connected pathways, and trapping metrics.

### 2.3 Image Processing and Analysis

All reconstructed micro-CT images were processed and analyzed using Thermo Fisher’s (Avizo) software. Initially, due to the 30% overlap between consecutive scans, images were registered to each other, followed by cropping to remove cone-beam artifacts. Subsequently, intensity normalization was performed across all sections to ensure consistent gray value distributions. Following these adjustments, the individual images were merged to reconstruct the complete sample. This procedure was repeated with all scans, registered and reassembled to the dry scan, providing a consistent reference for comparison across different saturation states. To reduce noise while preserving structural details, a nonlocal means filter was applied. Watershed segmentation was then implemented (Figure 4), and subtraction of the flow scans from the brine-saturated images was performed. The resulting non-wetting phase saturations were quantitatively compared, confirming the robustness and accuracy of the segmentation approach. Phase saturation was quantified by calculating the volume fraction of each segmented phase relative to the total pore space.

While watershed is a robust segmentation method, it is important to acknowledge that subtle changes in the gray scale histogram limits can impact connectivity analysis. It has been shown that choices in segmentation methodology can cause substantial variation in physics-based outputs.^35–37^ Numerically, this implies that even small misclassifications may induce errors in phase connectivity and non-wetting saturations on the order of 10–20%. These uncertainties can lead to under- or overestimation of residual trapping, saturation, connectivity, and permeability.^38^ The voxel size of 5.6 μm restricts the analysis to the macroporous fraction of the rock. Micropores and throats smaller than this threshold are unresolved, leading to underestimation of total porosity and permeability. However, because the observed flow remained within the capillary-dominated regime, and no evidence of invasion into unresolved pores was detected, the qualitative interpretation of displacement mechanisms and trapping behavior remains unaffected. Carrillo et al.^39^ showed that subresolution porosity can alter predictions of residual saturations by as much as 20% and relative permeability by a factor of up to 3, because unresolved porosity can act as persistent connectors between larger pores.

To analyze the pore-scale displacement mechanisms, we performed a pore network extraction on the segmented pore space of the rock. We used the open-source algorithm PoreXtractor^40^ to extract a skeletal network of pores and throats from the dry scan. This algorithm is based on a maximal inscribed sphere (maximal ball) method^41^ where it separates the void space into discrete pore bodies connected by throats. Each pore is characterized by the radius of the largest sphere that fits inside it, and each throat by the largest sphere that can pass through the constriction.^42^ The output of this analysis is a graph representation of the pore space, providing metrics such as pore size distribution, throat size distribution, and coordination number (the number of throats connected to each pore). We note that this network represents only the macroporosity of the sample (pores and throats with radius larger than ∼5.6 μm), any regions below that scale are not included and effectively treated as part of the solid matrix in this analysis. As a result, our network might under-represent the total porosity, permeability, and coordination number since subresolution pores and throats that could connect into the network were excluded.

Using the extracted pore network as a template, we then mapped the time-resolved fluid occupancy onto it. For each pore and throat identified in the network, we determined whether it was filled with decane or brine at the end of drainage and at the end of imbibition. This was done by overlaying the pore-network on the segmented fluid images from those time steps. A pore was classified as containing the non-wetting phase (decane) if more than 50% of its voxels were labeled as decane in the image, a threshold criterion based on previous pore-by-pore analyses.^43^ This approach avoids misclassifying pores that have only a thin film of brine or a minor presence of decane: it distinguishes pores that are predominantly filled with the non-wetting phase from those predominantly water filled. Similarly, throats were labeled by the majority phase present. Moreover, an estimation of the capillary entry pressures for some regions of rock were estimated using average throat diameters derived from pore network modeling and applying the Young–Laplace equation.

Finally, volume, surface area, count of distinct ganglia, and connectivity were derived from the images. This offers insights on how the non-wetting phase connectivity progressed.

## 3 Results and Discussion

### 3.1 Fluid Saturation Profiles and Phase Distribution

The saturation of brine and decane in the macro pores was quantified after each injection step. The fluid saturations were measured on segmented images of the whole sample both drainage (Figure 5a) and imbibition (Figure 5b). The non-wetting phase saturation profile increases systematically across the sample with rising non-wetting phase fractional flow. However, a local minimum in non-wetting saturation persists between 45 mm and 48 mm across all fractional-flow conditions (Figure 5a,b). Unlike other minor valleys that shift or diminish with changing fractional flow, this feature remains fixed in both position and amplitude, indicating that the non-wetting phase did not invade this region at any stage.

**Figure 5:**
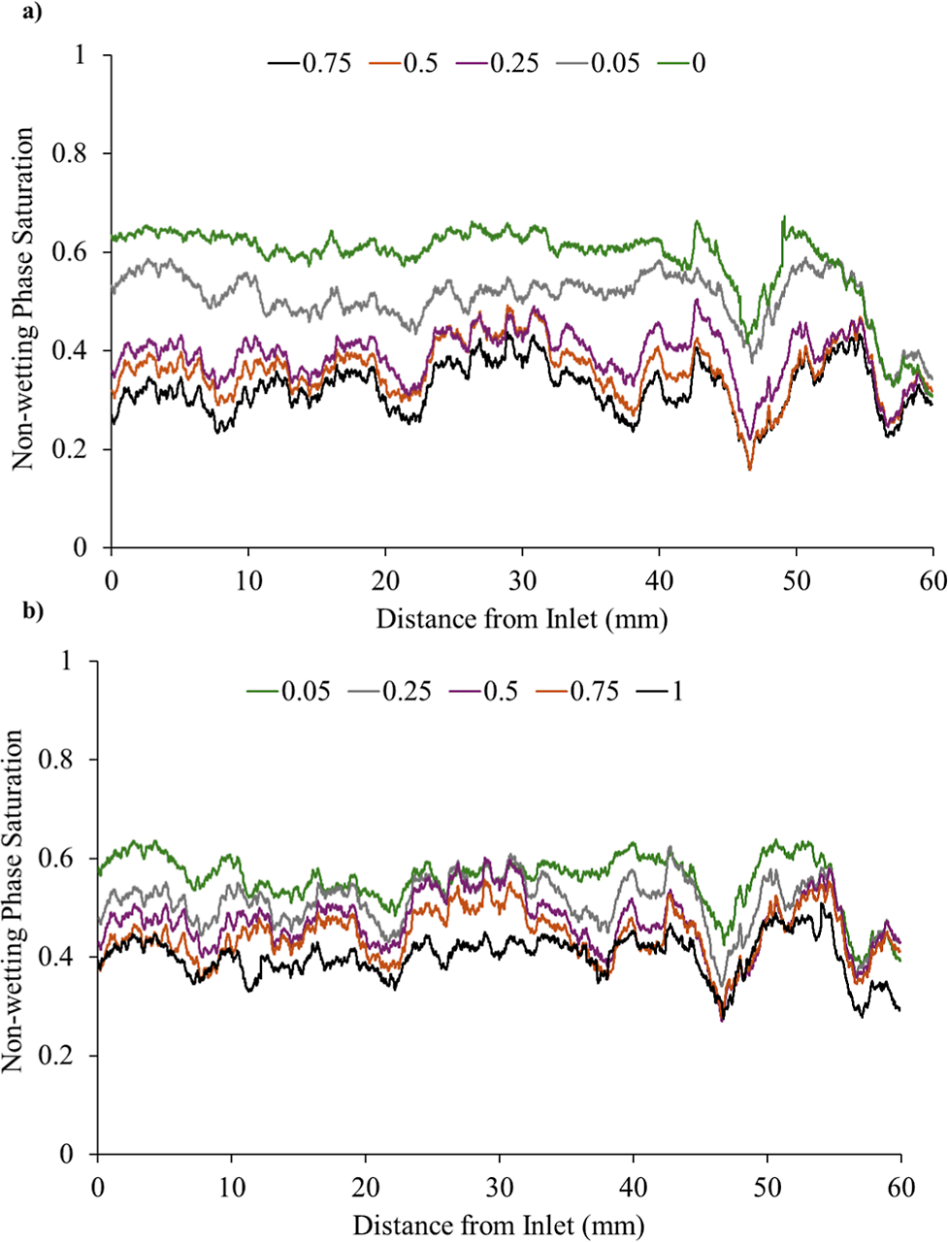
(a) 1D saturation profiles across the length of the sample during drainage (the legend indicates the imposed brine fractional flow). (b) 1D saturation profiles across the length of the sample during imbibition (the legend indicates the imposed brine fractional flow).

Table 1 presents the initial, intermediate, and residual saturations measured across the sample.

**Table 1:** Average Non-Wetting Phase Saturation During the Experiment

brine fractional flow (*f*_w_)	cycle	average non-wetting phase saturation
0.75	drainage	0.32
0.5	drainage	0.38
0.25	drainage	0.43
0.05	drainage	0.50
0	drainage	0.55
0.05	imbibition	0.54
0.25	imbibition	0.50
0.5	imbibition	0.47
0.75	imbibition	0.44
1	imbibition	0.41

A notable observation is that partial barrier region remains brine-saturated throughout the experiment and not invaded by the non-wetting phase even under 100% decane injection at the end of drainage. As shown in Figure 6, the tight downstream interval retains brine in both drainage (Figure 6a) and imbibition (Figure 6b), and demonstrates that decane could not enter those pores at any stage. This implies that the local capillary entry pressure in this zone exceeded the driving capillary pressure applied during the experiment. According to capillary pressure theory, smaller or poorly connected throats require higher threshold pressures (described by the Young–Laplace equation) for non-wetting phase invasion. Because this threshold was not reached, the pores remained brine-filled from start to finish, acting as a capillary barrier that blocked non-wetting advance and redirected flow along alternative pathways. This aligns with the findings of Pak et al.^44^ where they showed that relatively large pores connected by numerous small throats can act as capillary barriers, preventing invasion of the non-wetting phase and promoting local trapping.

**Figure 6:**
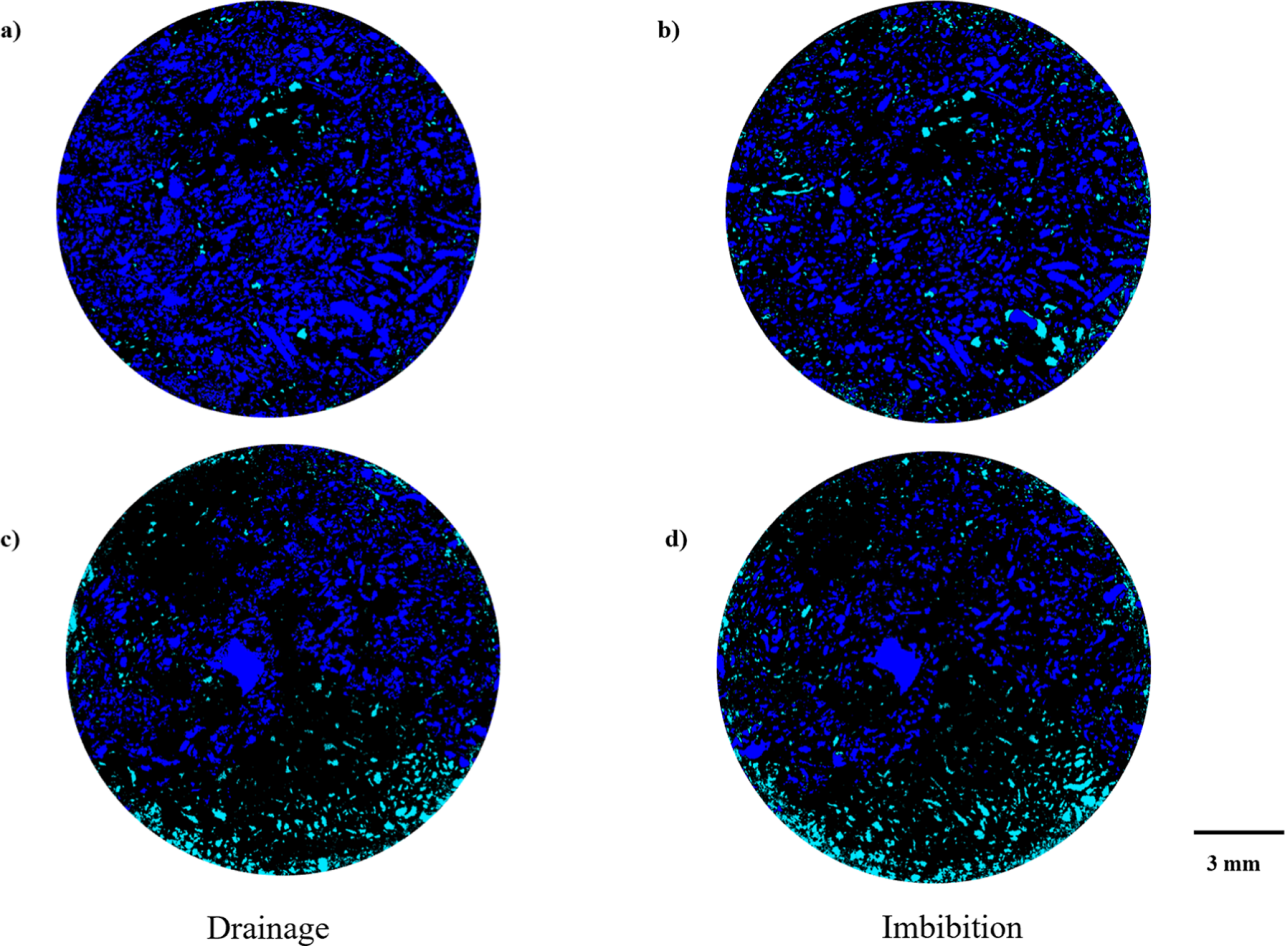
Representative 2D cross sections segmented images extracted from 3D micro-CT images at the end of drainage (*f*_w_ = 0) and imbibition (*f*_w_ = 1), captured from two distinct regions of the sample. Panels (a,b) correspond to a higher-porosity zone, while panels (c,d) are taken from a lower-porosity region, illustrating spatial variability in fluid distribution as a function of local porosity (black is grain, green is brine, and blue is decane).

Figure 7 provides a voxel-by-voxel view of sample where porosity and saturation are averaged in a 2 mm cube. The dry scan (Figure 7a) shows the spital distribution of the porosity profile, highlighting regions of higher and lower porosity. During drainage (Figure 7b), the non-wetting phase progressively fills the accessible pore space, accumulating at higher saturation upstream of the low-porosity zone, while the barrier region itself remains at low saturation. Moreover, at the end of the imbibition stage (Figure 7c) at full brine injection shows that while displacement occurs, a high residual saturation is clearly visible, confirming the combined roles of residual trapping and heterogeneity-driven capillary barriers in controlling fluid distribution.

**Figure 7:**
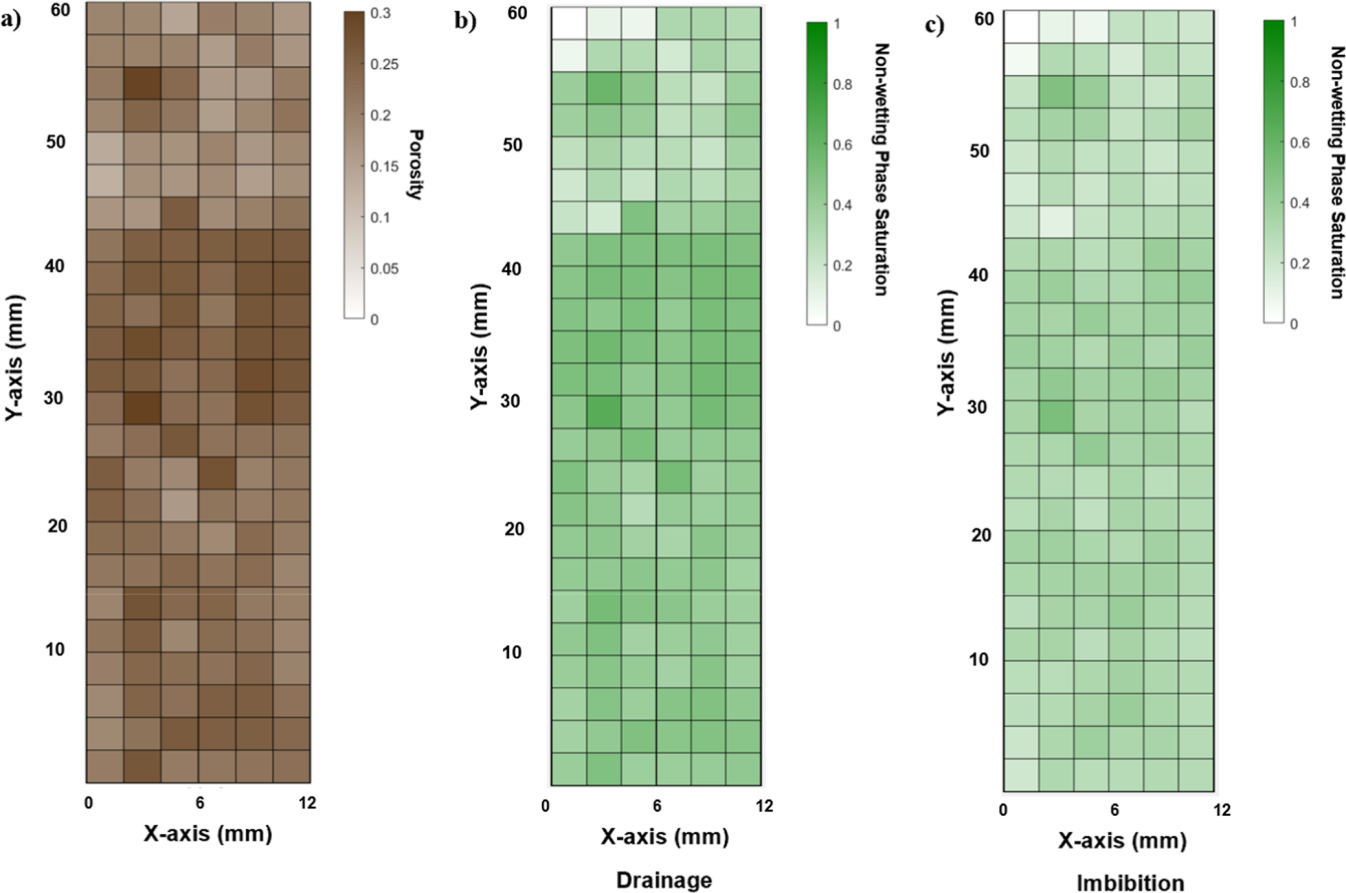
A voxel-by-voxel visualization of the sample along its central axis of the center-slice micro-CT images captured at key experimental stages: (a) dry scan, illustrating pore space distribution (porosity); (b) drainage, showing non-wetting phase (oil) saturation at a water fractional flow (*f*_w_ = 0); and (c) imbibition, displaying oil saturation at water fractional flow (*f*_w_ = 1).

### 3.2 Relative Permeability

Relative permeability measurements directly connects the pore-scale trapping mechanisms observed with their manifestation at the continuum scale. The hysteresis in relative permeability represents the macroscopic response to pore-scale processes such as snap-off, disconnection, and capillary barrier formation, demonstrating how internal heterogeneity governs both localized trapping and overall flow behavior.

The relative permeabilities obtained from drainage and imbibition cycles showed pronounced hysteresis in the non-wetting phase (Figure 8), alongside smaller hysteresis in the wetting phase, as typically expected in strongly water-wet porous media.^13^ At the end of the imbibition cycle, the non-wetting phase relative permeability shifts to the left (lower wetting phase saturation), indicating a reduction in relative permeability consistent with significant phase immobilization and enhanced residual trapping. The end point saturations further confirmed this interpretation, exhibiting a high residual non-wetting phase saturation. These findings align closely with those reported by Manoorkar et al.,^18^ who demonstrated an increased residual trapping in Edwards dolomite with initial and residual saturations of 28 and 20%, respectively. They also concluded that the flow behavior is strongly controlled by the heterogeneity across multiple flow rates.

**Figure 8:**
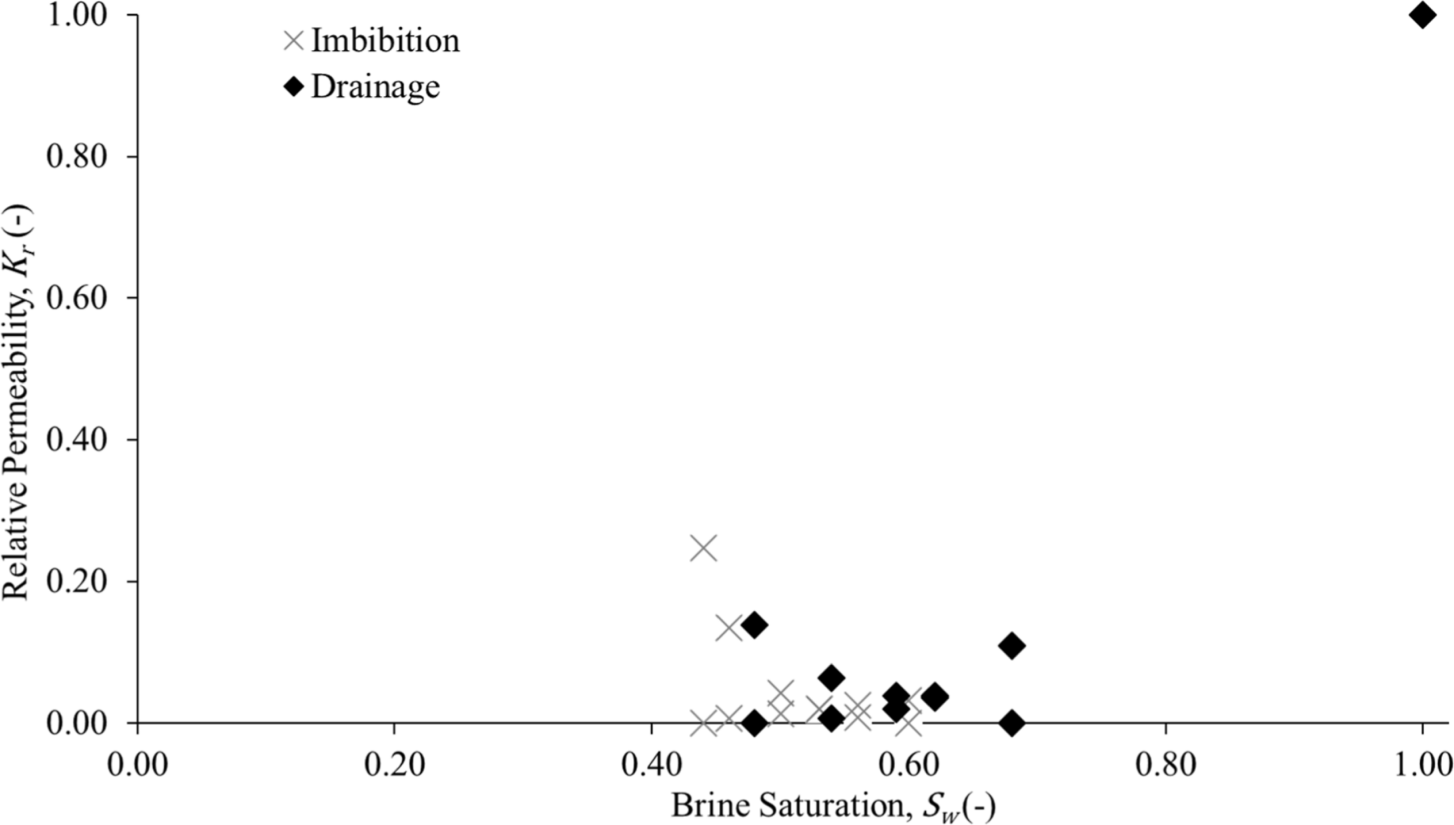
Relative permeabilities showing drainage and imbibition cycles for wetting and non-wetting phases.

### 3.3 Trapping Behavior

One of the primary objectives in geological CO_2_ storage is to maximize the extent of residual trapping namely, the amount of injected CO_2_ that becomes immobilized as disconnected ganglia once brine reoccupies the pore space.^2,45–48^ We assessed trapping efficiency by comparing the slice average non-wetting phase saturation along the sample at the end of drainage and imbibition. Across the full sample, a large proportion of the non-wetting phase introduced during drainage remained in place as isolated residual clusters following brine reinjection. Overall, the rock exhibited high volume of trapped non-wetting phase saturations with initial saturation of 55% and residual saturation of 41%.

Furthermore, a spatial variation in trapping efficiency was observed when comparing the bulk rock to regions adjacent to the partial capillary barrier (Figure 9). The barrier region exhibited a particularly high degree of trapping, with data points approaching the 1:1 line indicating that nearly all of the non-wetting phase initially present remained immobilized after imbibition. The slightly higher residual saturation immediately downstream of the barrier is attributed to the accumulation of disconnected non-wetting ganglia trapped against the barrier and retained during imbibition. The subsequent decrease in saturation toward the outlet reflects a capillary end effect, where reduced entry pressure at the open boundary facilitates enhanced brine invasion. This behavior is consistent with restricted phase accessibility due to finer pore structures and higher entry pressures in the barrier zone, leading to enhanced capillary trapping or build up behind the partial barrier.

**Figure 9:**
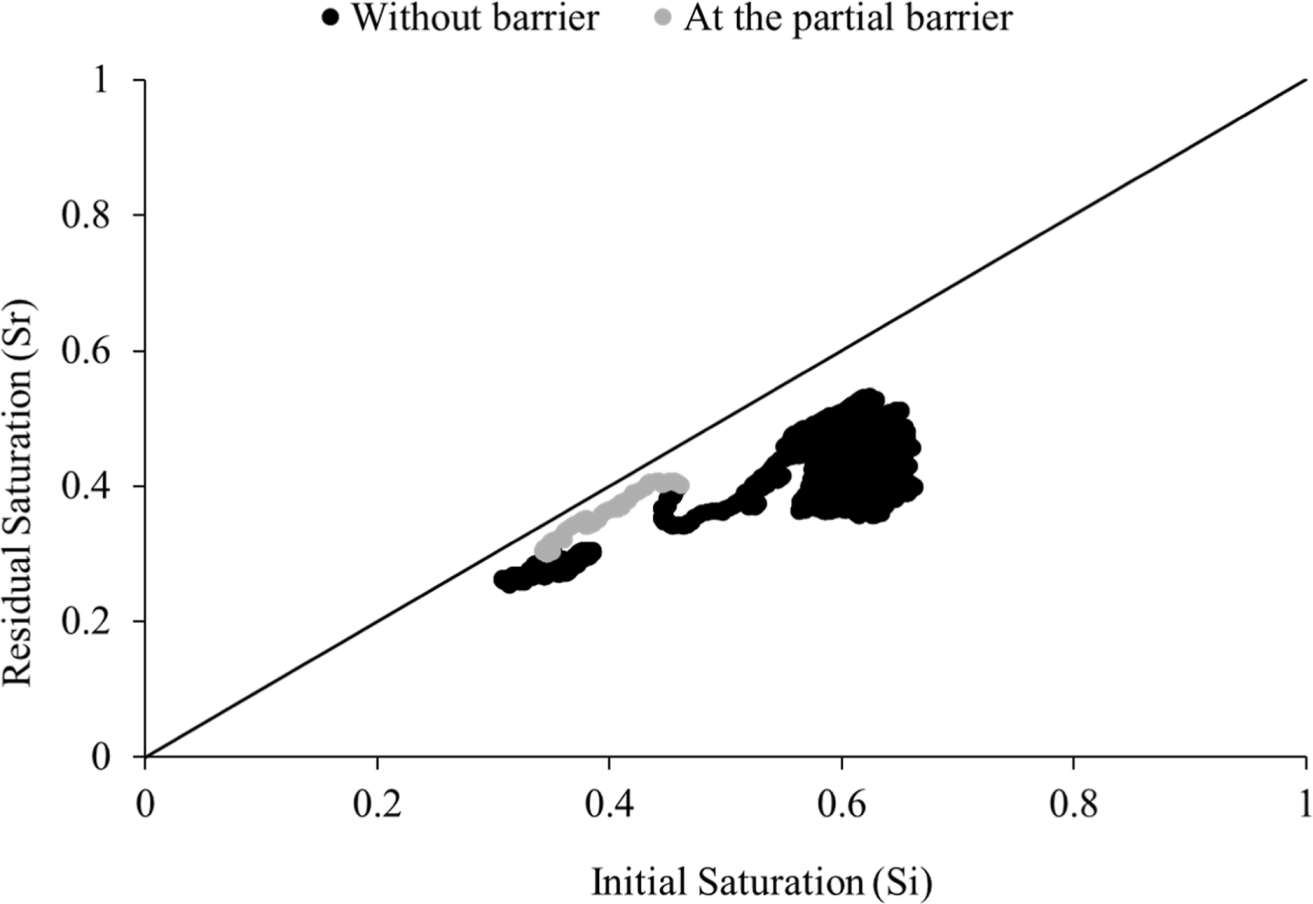
Slice average initial saturation versus residual saturation along the sample (gray is at the partial capillary barrier and black is the rest of the sample).

To further investigate the spatial characteristics of residual trapping, a ganglia analysis was conducted. After segmentation, each disconnected cluster of the non-wetting phase was uniquely labeled, allowing for quantitative analysis of ganglia count and spatial distribution, as shown in Figure 10. At the end of drainage, the pore space was dominated by a single large, connected non-wetting phase ganglion extending longitudinally, consistent with piston-like invasion behavior. However, in the vicinity of the partial barrier region, we observed a higher count of disconnected ganglia relative to other regions, which is not consistent with piston-like invasion. These observations indicate that the barrier region triggered early fragmentation of the invading phase, most likely due to locally elevated capillary entry pressures and limited pore connectivity. Roof snap-off could also account for such fragmentation, as it can generate either temporary or permanent isolation of the non-wetting phase depending on local geometry and pressure conditions.^32,49–51^ However, because no reconnection or coalescence of the phase was observed and given the strong correlation with the low-porosity barrier, the evidence points more convincingly to capillary thresholding and connectivity constraints rather than Roof snap-off as the dominant mechanism.

**Figure 10:**
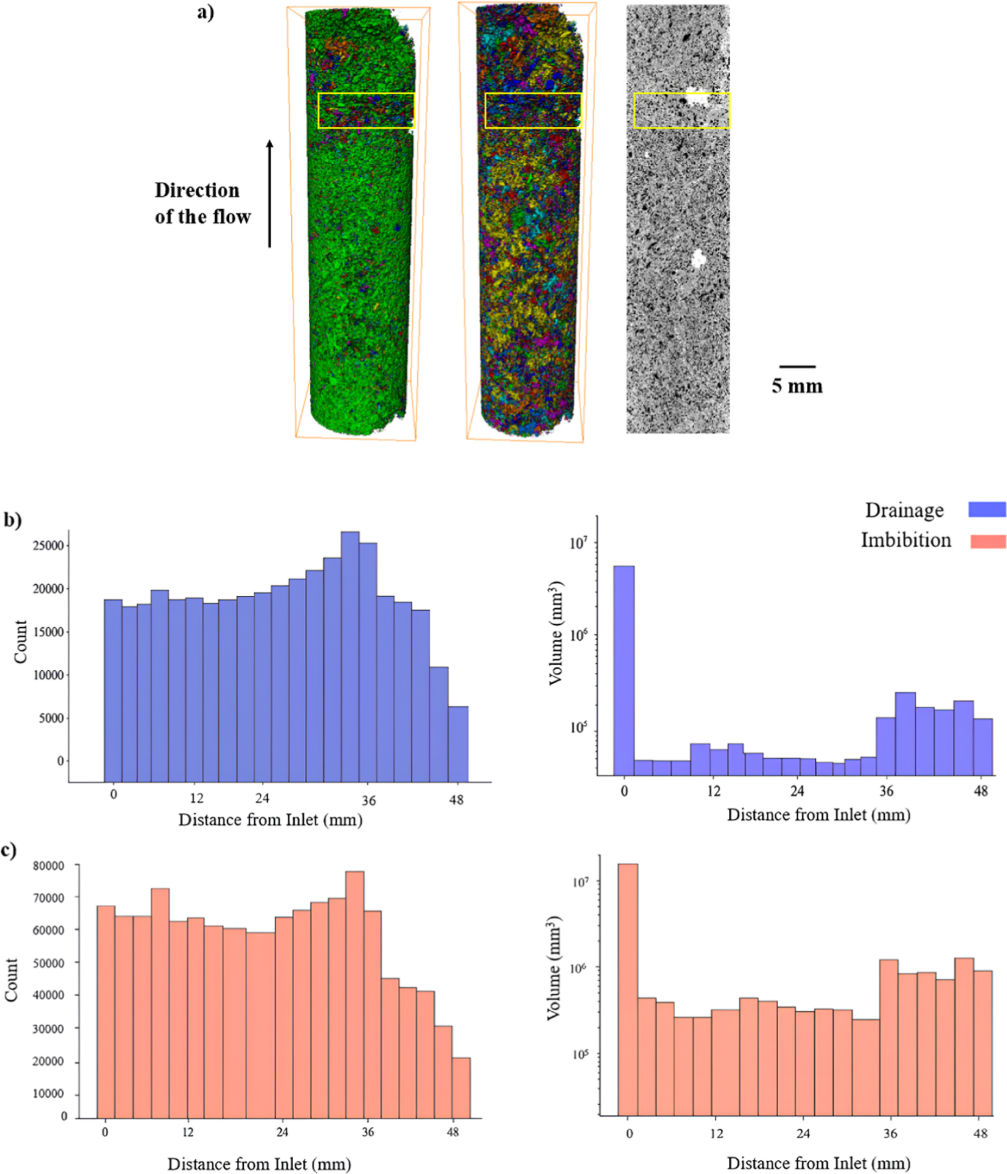
Analysis of non-wetting phase ganglia across the core after drainage and imbibition. (a) Three-dimensional renderings of the segmented non-wetting phase, superimposed on greyscale micro-CT images of the rock matrix. The image on the left shows the distribution at the end of drainage, where a single large, continuous ganglion (green) spans most of the sample length, with smaller disconnected clusters appearing near the barrier. The image on the right shows the distribution after imbibition, where the non-wetting phase has fragmented into multiple smaller ganglia. Each disconnected ganglion is assigned a unique color for clarity. The greyscale image shows the dry image, included to give spatial reference of the barrier region. (b) Histograms showing the number of ganglia (left) and their volume (right) along the core length at the end of drainage. (c) Histograms showing the number of ganglia (left) and their volume (right) along the core length at the end of Imbibition. Figure b highlights an increase in the number of disconnected clusters away from the barrier region and retention of ganglia count around the barrier region after brine reinvasion. While figure c illustrates the breakdown of the large ganglia at the end of drainage to smaller ones after imbibition.

Following imbibition, the single large ganglion had broken down into numerous smaller ganglia distributed more uniformly throughout the sample. This increase in ganglia count reflects the onset of the snap-off trapping mechanism during brine reinvasion.^51,52^ Notably, after imbibition in the barrier region, the ganglia count remains almost similar to that of the drainage.

These observations support the findings from the trend of residual saturation as a function of initial saturation, Figure 9, that the barrier region acts to retain the non-wetting phase behind it, promoting early disconnection and enhancing the overall trapping efficiency. Hence, residual trapping in this sample arises from two distinct mechanisms: pore-scale snap-off and build-up of the non-wetting phase behind the capillary barrier.

### 3.4 Pore Network Occupancy and Connectivity

The pore network modeling and occupancy analysis^43,53^ was performed on the sub-volumes of equal size across the length of the sample (Figure 11a). The sub-volumes are selected at the barrier region and away from the barrier to be able to make a direct comparison of the connectivity and displacement patterns. The resolved throat size distribution within the barrier region exhibits narrower throats compared to the upstream region away from the barrier zone (Figure 11b).

**Figure 11:**
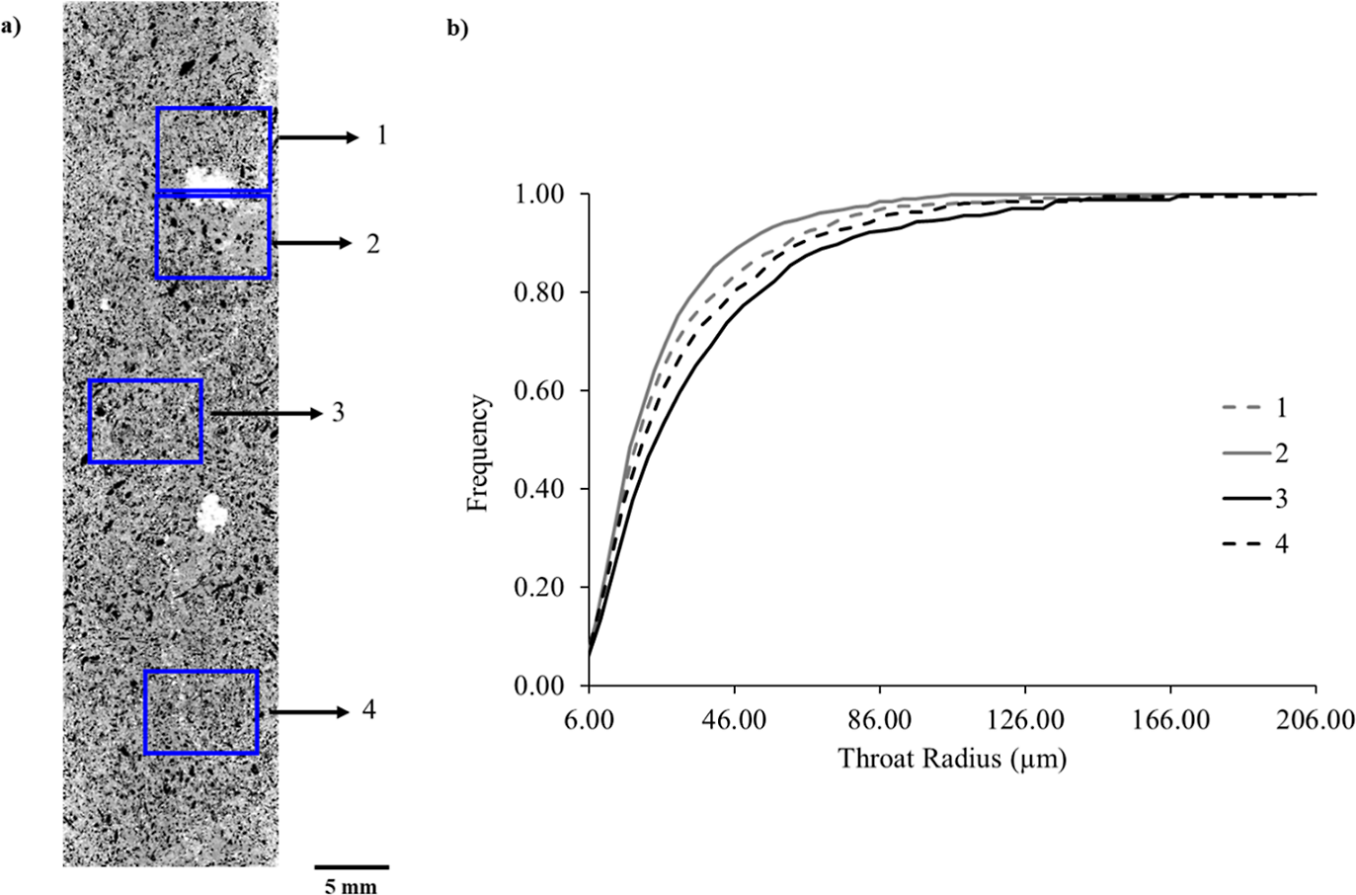
(a) Spatial distribution of selected sub-volumes across the sample, used for detailed analysis. (1,2) denotes the barrier zone characterized by reduced porosity and distinct flow behavior; (3,4) corresponds to a higher porosity zone located away from the heterogeneity barrier. (b) throat-size distribution of sub-volumes (1–4).

The detailed pore-by-pore occupancy analysis during drainage and imbibition provides direct link between the entry capillary pressure (controlled by throat size) to displacement behavior. Figure 12 illustrates the evolution of the non-wetting phase occupancy in the selected sub-volumes both in pores and throats, respectively. During drainage and subsequent imbibition, the barrier regions (1 and 2) exhibit minimal changes saturation between the two flow stages in pores, while in throats we only see the changes in the smaller throats indicating limited fluid displacement and a high degree of residual non-wetting phase retention. This minor apparent changes in occupancy occur near the barrier boundaries where slightly larger throats are connected to smaller pore clusters or where the imaging resolution approaches the voxel limit. These do not represent full invasion but rather marginal redistribution within the partially resolved pore space. In contrast, the sub-volumes located away from the barrier (3 and 4) display more pronounced changes in phase occupancy, with larger displacement during imbibition. This contrast shows the influence of local heterogeneity and connectivity in phase displacement and trapping efficiency.

**Figure 12:**
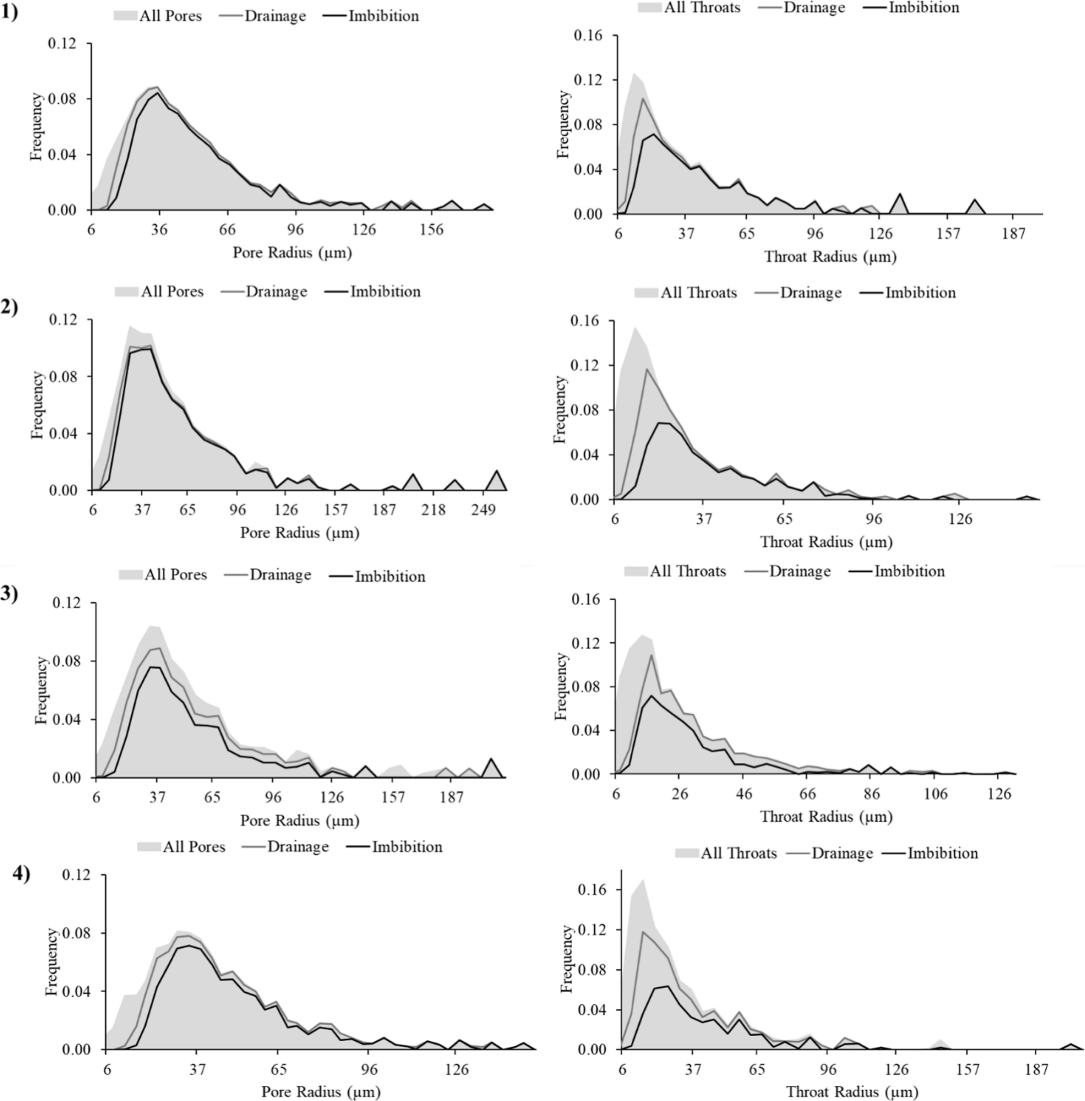
Pore and throat occupancy analysis comparing barrier and non-barrier regions. Panels (1–4) correspond to sub-volumes (1–4), respectively shown in Figure 11.

Two key pore-network metrics are the coordination number and the pore–throat aspect ratio. The coordination number, defined as the number of throats connected to a given pore,^32^ quantifies network connectivity and directly impacts simulated flow behavior and saturation patterns.^54^ In well-sorted, high-permeability sandstones such as Bentheimer or Berea, the mean coordination number is typically 4–6, reflecting a highly interconnected structure.^41,55^ In contrast, the Edwards Brown dolomite exhibits a lower connectivity, with a mean coordination number of approximately 2; over 30% of the resolved pore bodies are connected by only two or fewer throats (Figure 13a). However, it is important to acknowledge the uncertainty introduced by imaging resolution; subresolution throats may provide connections that are not visible in the images. However, no evidence of non-wetting invasion into such unresolved features was observed during the experiment. If the viscous pressure gradients during the experiment had been sufficient to overcome entry pressure of those, the non-wetting phase could have been seen in the “isolated” pores. Since this was not the case, it implies that those connections did not activate, and these pores remain occupied by brine.^56^

**Figure 13:**
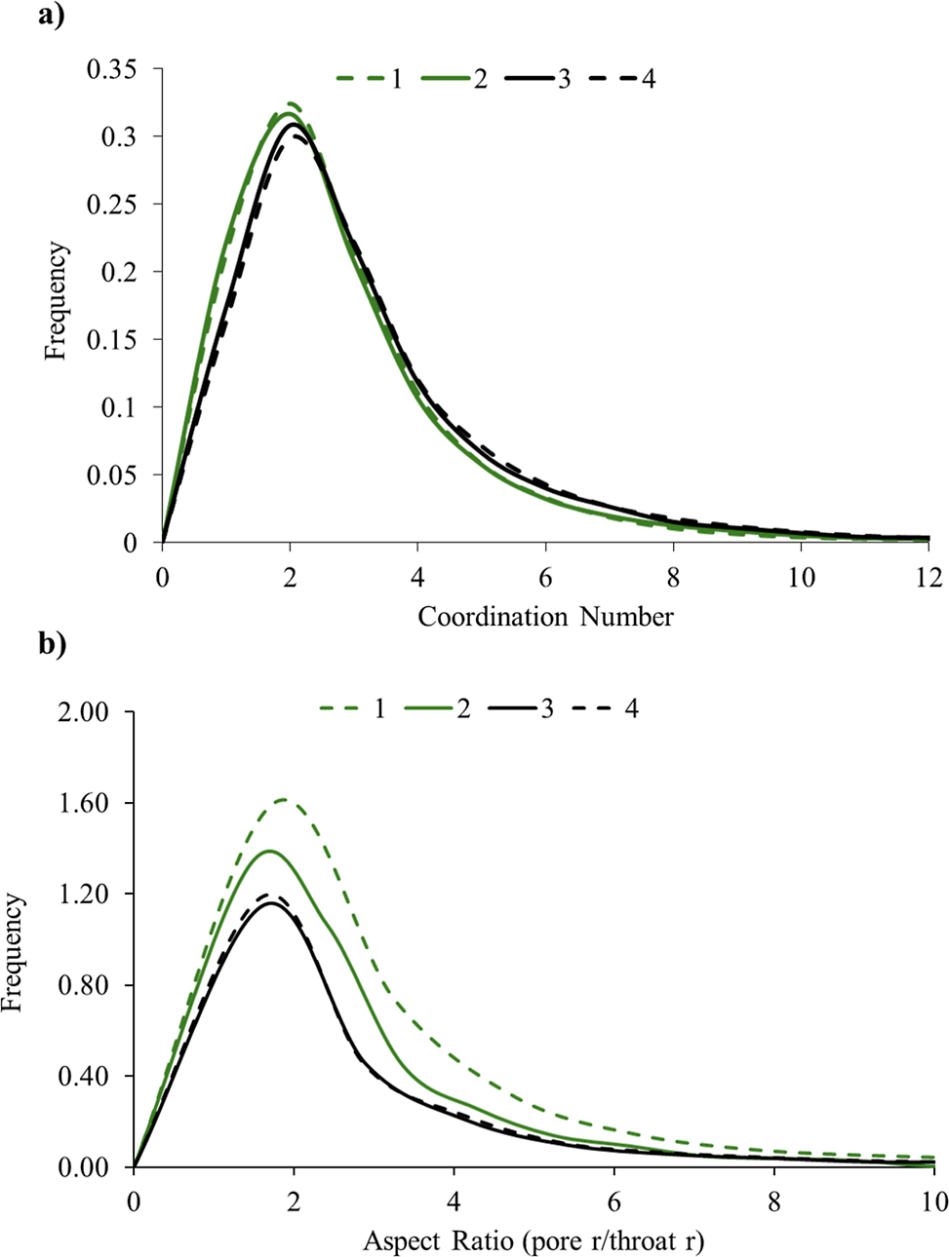
(a) Distribution of coordination number and (b) aspect ratio. These distributions are evaluated for the four sub-volumes shown in Figure 11.

The second metric and the more important one, is aspect ratio, defined as the ratio of pore radius to the average radius of its connecting throat(s).^32^ A higher aspect ratio indicates large pores connected by narrower throats, a geometry that strongly favors snap-off during imbibition. In the Edwards Brown dolomite, the barrier region shows higher aspect ratios compared to the other regions, implying an increased likelihood of snap-off and enhanced residual trapping.

### 3.5 Entry Capillary Pressure

The Young–Laplace equation provides a quantitative basis for estimating capillary entry pressures as a function of pore throat diameter, interfacial tension, and contact angle. For our water–decane system with an interfacial tension of approximately 47 mN/m, a throat diameter of 5 μm corresponds to an entry pressure near 0.02 MPa, comfortably within our imposed flow conditions. In contrast, throats in the 0.6 μm range would require entry pressures exceeding 0.3 MPa and remained sealed with brine during our experiment.

Upon analyzing the average throat sizes and applying the Young–Laplace equation to the four sub-volumes, we observed that the barrier region exhibits higher capillary entry pressures than the areas away from it as shown in Figure 14.

**Figure 14:**
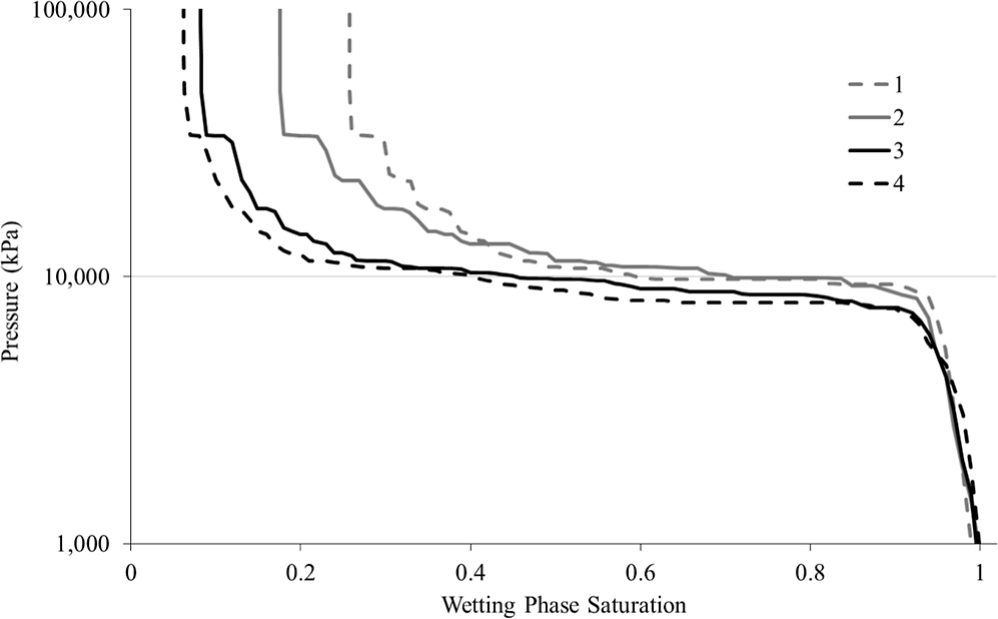
Entry capillary pressure in the barrier region and away from the barrier. The four sub-volumes shown in Figure 11 are analyzed.

As mentioned earlier, the four sub-volumes analyzed are equal in size and image resolution. Under such conditions, it is expected to have comparable number of resolved pores and throats across the selected regions. The extracted networks show that the resolved pore-body radii range from approximately 6 to 60 μm and throat radii from ∼5.6 to 27 μm across the four sub-volumes (Table 2). Throats smaller than one voxel in radius (≈5.6 μm) are below the detection threshold and thus not explicitly represented in the analysis. In the barrier sub-volumes, the number of identified pore bodies and connecting throats was nearly halved relative to the regions away from the barrier (Table 2). This suggests that a substantial portion of the pore space in the barrier falls below the resolution threshold of our imaging. As a result, the calculated capillary entry pressures in this region are likely underestimated, since unresolved pores and throats smaller than the voxel size would require even higher entry pressures to be invaded. Therefore, the true capillary threshold in the barrier is expected to be higher than what is inferred from the resolved structure.

**Table 2:** Summary of the Pore Network Analysis for the Four Sub-Volumes (Barrier Region and Away from the Barrier)

sub-volume	porosity (%)	permeability (mD)	number of pores (10^3^)	number of throats (10^3^)	pore radius range (μm)	throat radius range (μm)	average entry pressure (kPa)
barrier (1)	17	12	41	40	6–42	5.6–18	12
barrier (2)	19	13	45	42	7–45	5.6–19	11
away from barrier (3)	24	103	68	76	8–58	6–25	9
away from barrier (4)	25	112	70	80	9–60	6–27	9

This interpretation is reinforced by the absolute permeability measurements from the pore network, Table 2, shows that the barrier region exhibits values nearly an order of magnitude lower than those measured in the higher-porosity zones. Taken together, these observations indicate the presence of a smaller, more resistive pore structure within the barrier and highlights the critical role of local pore structure in controlling phase accessibility and flow.

The analysis shows that enhanced trapping is controlled by a combination of factors, with capillary entry pressure, coordination number, and pore–throat aspect ratio playing the most significant roles. Elevated entry pressures limit invasion across the barrier, coordination number shows a limited connectivity, and high aspect ratios favor snap-off during imbibition, together producing the persistent immobilization observed in this sample.

One of the main observations is that digital rock workflow provides a practical way to evaluate these mechanisms directly from micro-CT images. Regions of reduced porosity can be mapped as potential barriers, and pore-network analysis offers further diagnostics. Aspect ratios highlight where snap-off is likely to occur, coordination number gives an indication on connectivity, while capillary pressure functions capture threshold behavior and the extent of capillary trapping. Taken together, these metrics describe the two complementary components of the trapping process—snap-off, governed by geometry and connectivity, and capillary thresholding, governed by entry pressure contrasts. Flow experiments remain valuable for confirmation, particularly in clarifying the role of sub-resolution porosity, since displacement patterns provide evidence of whether hidden connections contribute to fluid migration or remain inactive.

### 3.6 Implications for Larger Scale Flow

The central uncertainty in extrapolating from pore-scale observations is how connectivity within the imaged domain continues into larger volumes beyond the micro-CT sample. A single centimeter-scale plug captures only a fraction of the connectivity architecture that governs plume migration in the reservoir. Whether the barriers and low-connectivity zones observed at the pore scale persist, merge, or average out at larger scales remains an open question, but their impact must be considered when linking pore-scale metrics to reservoir-scale parameters.

A second consideration is the difference in pressure regimes between laboratory and field conditions. In the core-flood experiments presented here, the viscous pressure drop across the 6 cm plug is relatively small, meaning that invasion is dominated by capillary thresholds and limited to the most accessible pathways. In the field, however, a plume migrating over tens to hundreds of meters will experience larger viscous pressure gradients and gravity–capillary interactions that can overcome additional entry pressures. This raises the possibility that more pores may be invaded under reservoir conditions than are accessed in the laboratory. Manoorkar et al.^18^ provide supporting evidence, showing that increasing viscous forces by an order of magnitude accessed additional pore space and modestly shifted initial saturations from 28 to 33%. Importantly, while this effect demonstrates that field-scale forces can mobilize some additional pore space, the overall trapping efficiency remained high, confirming that heterogeneity-driven barriers continue to exert a strong influence even under more favorable displacement conditions.

Together, these observations suggest that pore-scale diagnostics such as coordination number, aspect ratio, and capillary entry pressure provide a lower-bound estimate of trapping potential. Field conditions may mobilize some otherwise isolated pores, but the persistence of barriers and snap-off geometries means that heterogeneity-driven trapping remains a controlling factor at reservoir scale.

In summary, upscaling pore-scale heterogeneity into continuum-scale models requires translating geometric and topological features into effective constitutive parameters. The metrics derived here (coordination number, pore–throat aspect ratio, and entry pressure) can be embedded in models to represent local variations in connectivity. For example, locally elevated capillary entry pressures associated with barrier zones can be represented as sub-grid-scale contrasts in the entry-pressure field, which in turn govern plume fingering and trapping at the Darcy scale. Similarly, coordination number and aspect ratio statistics can be used to define effective residual saturation functions or trapping coefficients that vary spatially according to heterogeneity intensity.

Beyond parametrization, these observations also motivate stochastic or multiscale modeling approaches, in which pore-scale heterogeneity is explicitly sampled to inform the statistical structure of continuum models. Such approaches have already been applied to sandstones with layered heterogeneity, but comparable efforts for carbonate systems remain limited.^57^

## 4 Conclusions

This study examined the influence of pore-scale heterogeneity on multiphase flow behavior and trapping mechanisms in an Edwards Brown dolomite sample under capillary-dominated conditions representative of the capillary limit relevant to geological CO_2_ storage. Using micro-CT imaging we directly visualized fluid configurations during drainage and imbibition, capturing the effects of local pore structure on saturation distribution, fluid accessibility, and residual trapping.

The sample exhibited substantial heterogeneity both in the overall structure and in the upstream region the acts as a partial capillary barrier. This region resisted invasion by the non-wetting phase during drainage, even at high fractional flow, leading to an accumulation of the non-wetting phase upstream. Moreover, the mean coordination number of roughly two indicates limited pore connectivity, which restricts fluid mobility, while the high pore–throat aspect ratios in the barrier region further promote early snap-off during imbibition. Together, these factors explain the residual trapping observed in the system (initial saturation 55%, residual saturation 41%), which arises from a combination of pore-scale snap-off and accumulation of the non-wetting phase behind the capillary barrier.

These findings stress the relevance of recognizing and incorporating pore-scale heterogeneity in the design and modeling of CO_2_ storage operations. While such heterogeneity can enhance trapping and contribute to long-term containment, it can also limit accessible pore volume and skew estimates of plume migration if not properly accounted for. Conventional models that treat carbonate formations as homogeneous media risk oversimplifying this complexity, potentially underestimating both residual trapping and flow diversion. As seen from this rock, the increased trapping observed in the barrier region is best explained as a combined effect of multiple factors. In this setting, pores are either not invaded or invaded but quickly disconnected as the non-wetting phase is pinched off, a behavior that is captured by the elevated pore–throat aspect ratios measured in the barrier. This implies that predictive models cannot rely solely on a Darcy-scale description, since a standard drainage capillary pressure alone does not capture the enhanced trapping. Instead, pore-scale diagnostics such as aspect ratio and coordination number are needed to explain and predict the observed immobilization.

Methodologically, this study demonstrates the power of combining pore-scale imaging with dynamic displacement experiments to investigate the effect of heterogeneity on fluid flow in complex porous media. At the same time, the work highlights current limitations in segmentation and resolution constraints, which introduce uncertainty into network-based metrics such as coordination number. Continued advances in image processing and multiscale characterization are needed to resolve these ambiguities and support more accurate pore-to-field scale translation.

The use of *n*-decane as an analogue for CO_2_ under ambient conditions likely results in higher apparent residual saturations than would be observed with supercritical CO_2_ at reservoir pressure and temperature. This arises from the higher interfacial tension and higher viscosity of *n*-decane relative to CO_2_, which suppress viscous instabilities and enhance capillary immobilization. Consequently, while the absolute saturation values reported here should not be interpreted as direct quantitative predictions for CO_2_, the displacement and trapping mechanisms we observe are representative of those that occur under capillary-dominated flow in heterogeneous formations.

At the same time, our results provide a mechanistic foundation for upscaling. The pore-scale observations of barrier-induced trapping and limited connectivity can be translated into continuum models through parameters such as effective capillary entry pressure distributions, aspect ratio–controlled snap-off functions, and local trapping coefficients. These findings highlight that heterogeneity exerts a first-order control on plume migration and immobilization efficiency, and must be explicitly incorporated into large-scale simulations of CO_2_ storage in carbonate systems. This study demonstrates how internal heterogeneity governs the structure and persistence of residual trapping and provides direct pore-scale evidence of its controlling influence on subsurface flow and storage security.

Overall, the results contribute to a more nuanced understanding of how fine-scale rock features impact CO_2_ storage performance. By explicitly accounting for internal barriers, and limited connectivity, future reservoir models can better reflect the physical realities of carbonate systems. This will enable more robust predictions of plume behavior, more informed risk assessments, and ultimately, more secure long-term carbon storage.

## Supplementary Material



## Data Availability

The segmented images used in this study can be downloaded from here: darraj, Nihal (2025). Heterogeneity Driven Trapping at the Pore-Scale in Edwards Brown Dolomite. figshare. Data set. 10.6084/m9.figshare.30510944.v1.

## Notes

Additional details of the mercury intrusion porosimetry data, the image segmentation procedure, and the distribution of saturation in different parts of the sample. Supplemental analysis of the pressure signal using wavelet methods to evaluate the strength of the displacement events (PDF)
