# Supplementary material for: Heterogeneity Driven Trapping at the Pore-Network Scale in Edwards Brown Dolomite
Source: Energy Fuels. 2025 Dec 16;40(1):528–42. doi: 10.1021/acs.energyfuels.5c04544 (PMC12797232; doi:10.1021/acs.energyfuels.5c04544)
Supplement: Supplementary file 1 [file ef5c04544_si_001.pdf]

Heterogeneity Driven Trapping at the Pore-Network Scale in Edwards Brown Dolomite

Supplementary information

Nihal Darraj[1]*, Sojwal Manoorkar[2], Catherine Spurin[3], Sajjad Foroughi[1], M Saleh[1], Steffen Berg[4], Martin J. Blunt[1], and Samuel Krevor[1]

[1] Department of Earth Science and Engineering, Imperial College London, SW7 2AZ, London, UK

[2] Department of Geology, Ghent University, Krijgslaan 281, 9000 Ghent, Belgium

[3] Energy Science and Engineering, Stanford University, 94305, Palo Alto, USA

[4] Shell Global Solutions International B.V., Grasweg 31, 1031 WG, Amsterdam, Netherlands

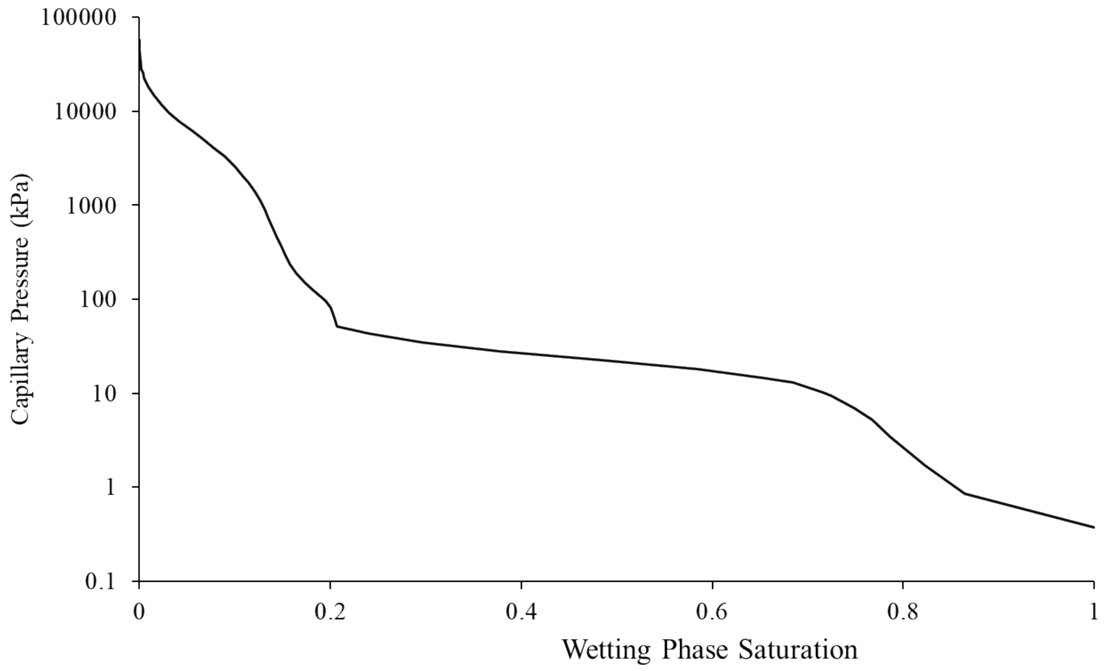

Figure S1: Mercury invasion capillary pressure (MICP). It highlights multiple entry pressure values.

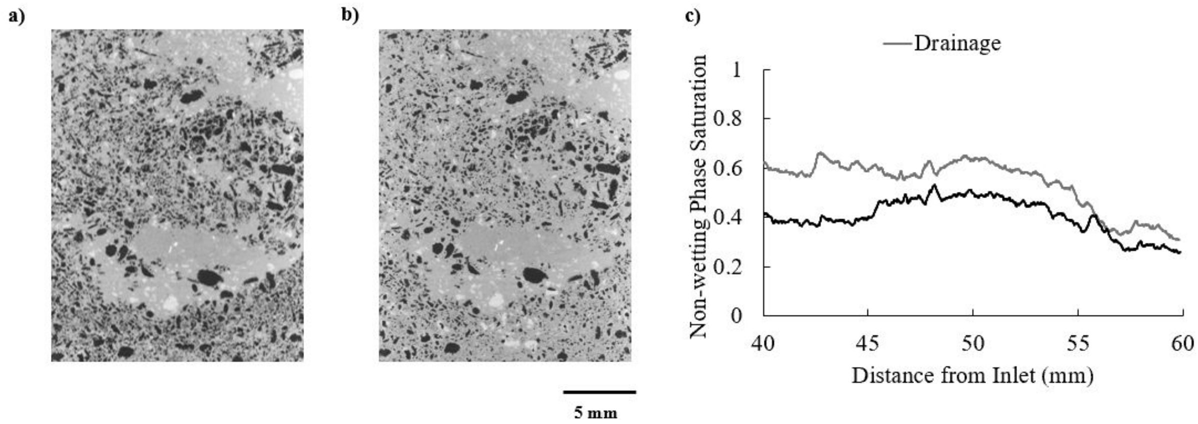

Figure S2: Two-dimensional cross-sections from a three-dimensional micro-CT image, zoomed in on the downstream heterogeneous region of the core. Panel (a) shows the fluid distribution at the end of drainage, while panel (b) captures the same region at the end of imbibition. Panel (c) displays the corresponding one-dimensional non-wetting saturation profile, illustrating the contrast in saturation before and after the heterogeneity. Notably, the non-wetting phase saturation remains unchanged within the heterogeneous zone, indicating limited displacement.

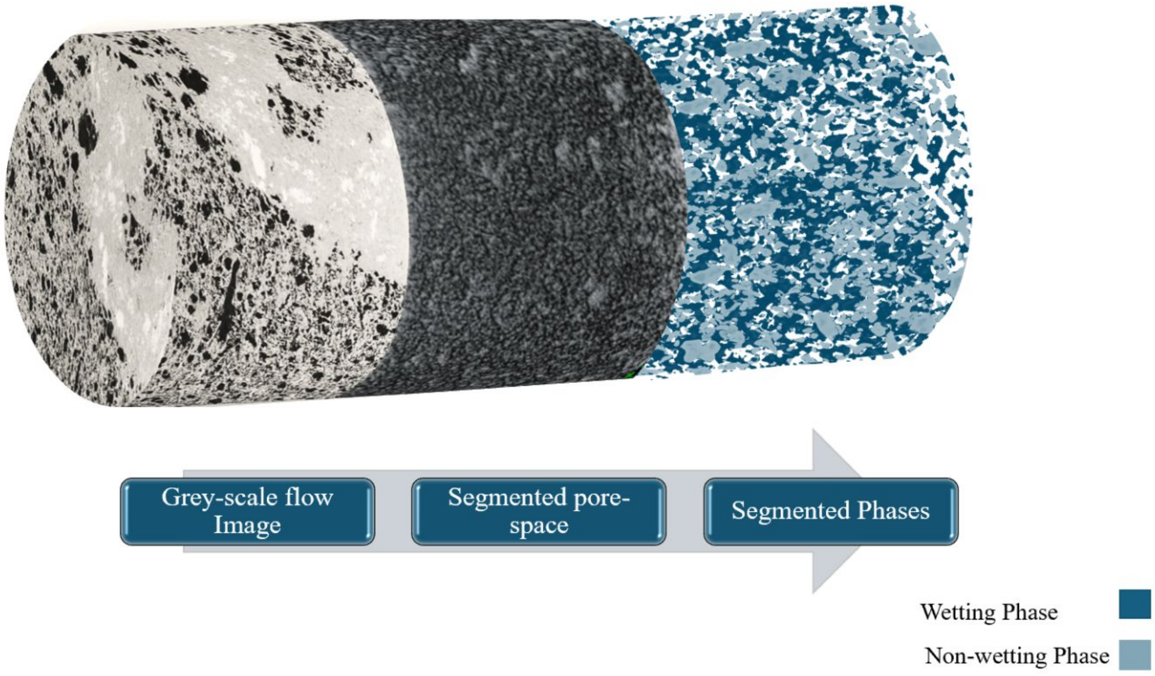

Figure S3: Segmentation workflow visualised for Edwards Brown dolomite. sandstone. After imaging, the grey-scale image, segmented to separate pore space and grain phases. Finally, the fluid phases are segmented into wetting (dark blue) and non-wetting (light blue) phases and then calculated from the total pore-space to determine the saturation.

Additional Analysis

Wavelet analysis provides a powerful tool to capture transient pore-scale dynamics, such as Haines jumps and the swelling of wetting layers, which are often obscured in averaged signals (Spurin et al., 2023). By decomposing pressure data into time–frequency space, discrete displacement events can be isolated and their intensity quantified. In our experiments, imbibition is associated with notably higher-pressure fluctuations and stronger dynamic events compared to drainage, consistent with the increased frequency of snap-off and enhanced trapping. Figures 4 through 7 shows the wavelet analysis of the pressure signal at drainage and imbibition for the same fractional flow.

It is notable that, when combined with dynamic imaging, wavelet analysis can provide a direct link between observable pore-scale events and pressure fluctuations, thereby bridging discrete displacement dynamics with macroscopic flow responses.

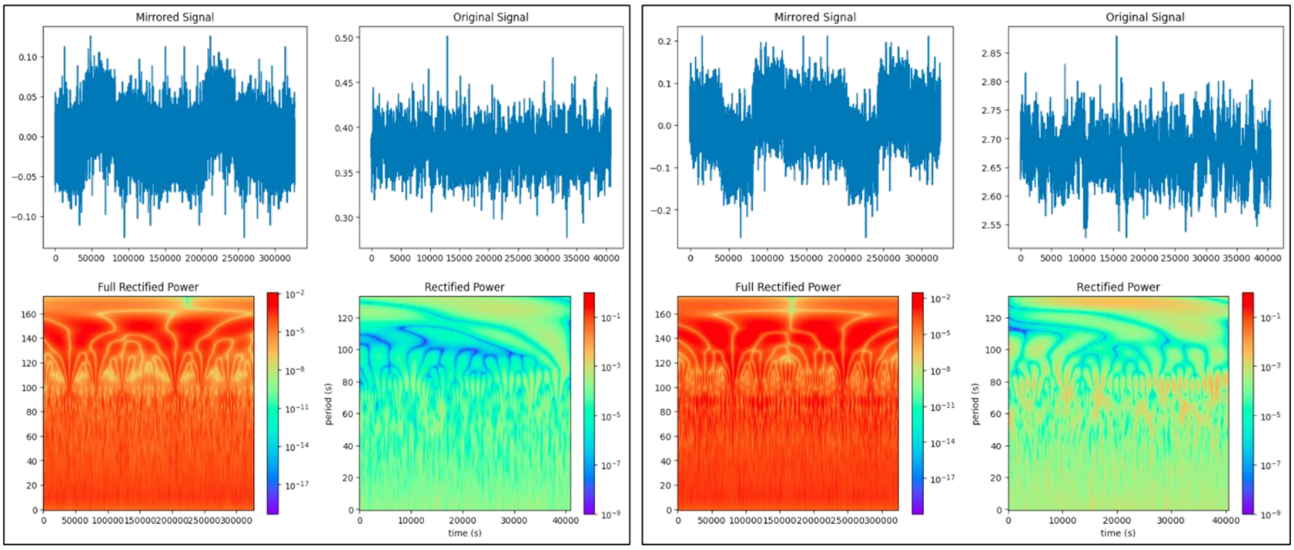

Drainage Imbibition

Figure S4: Wavelet analysis for brine fractional flow of 100% (f_w=1).

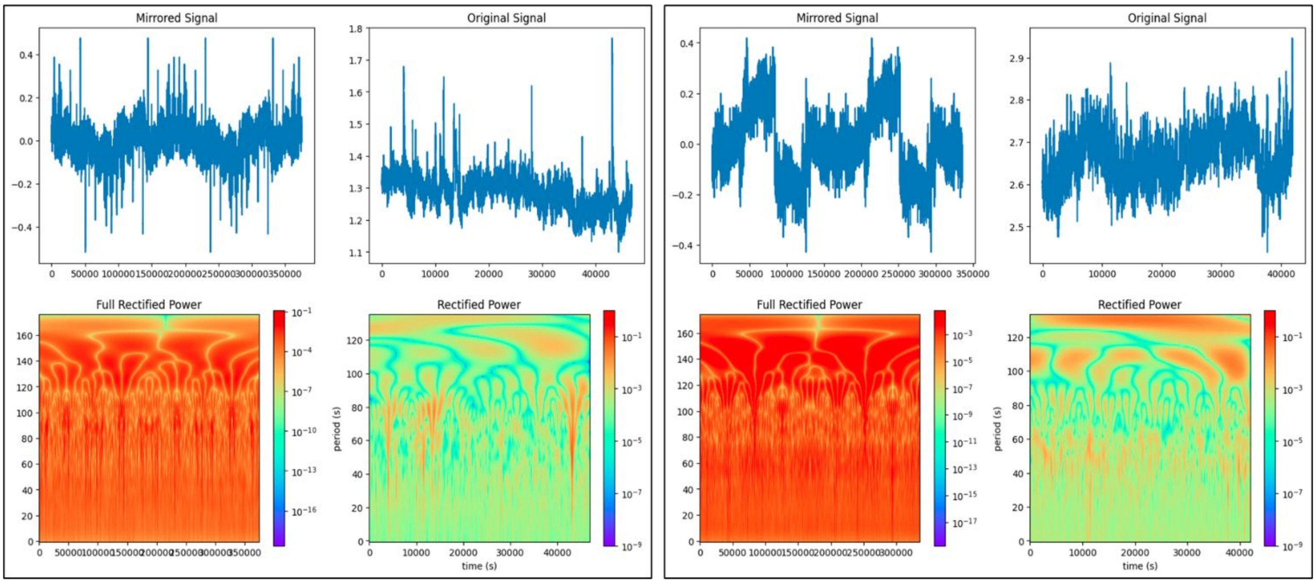

Drainage Imbibition

Figure S5:Wavelet analysis for brine fractional flow of 75% (f_w=0.75).

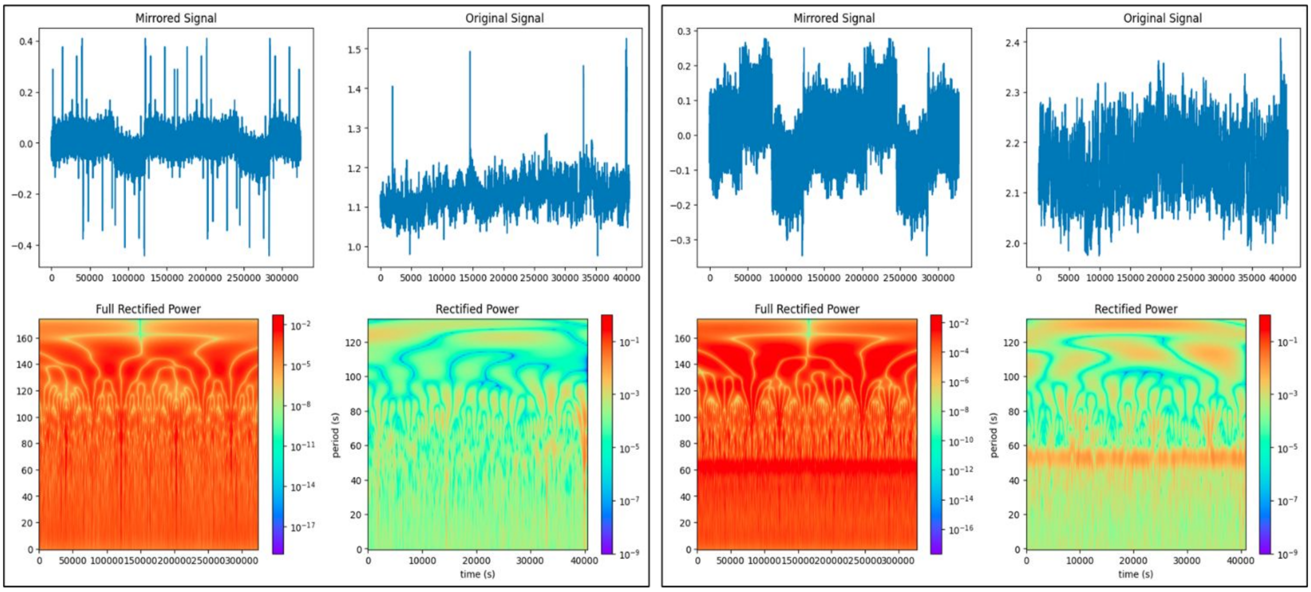

Drainage Imbibition

Figure S6: Wavelet analysis for brine fractional flow of 50% (f_w=0.5).

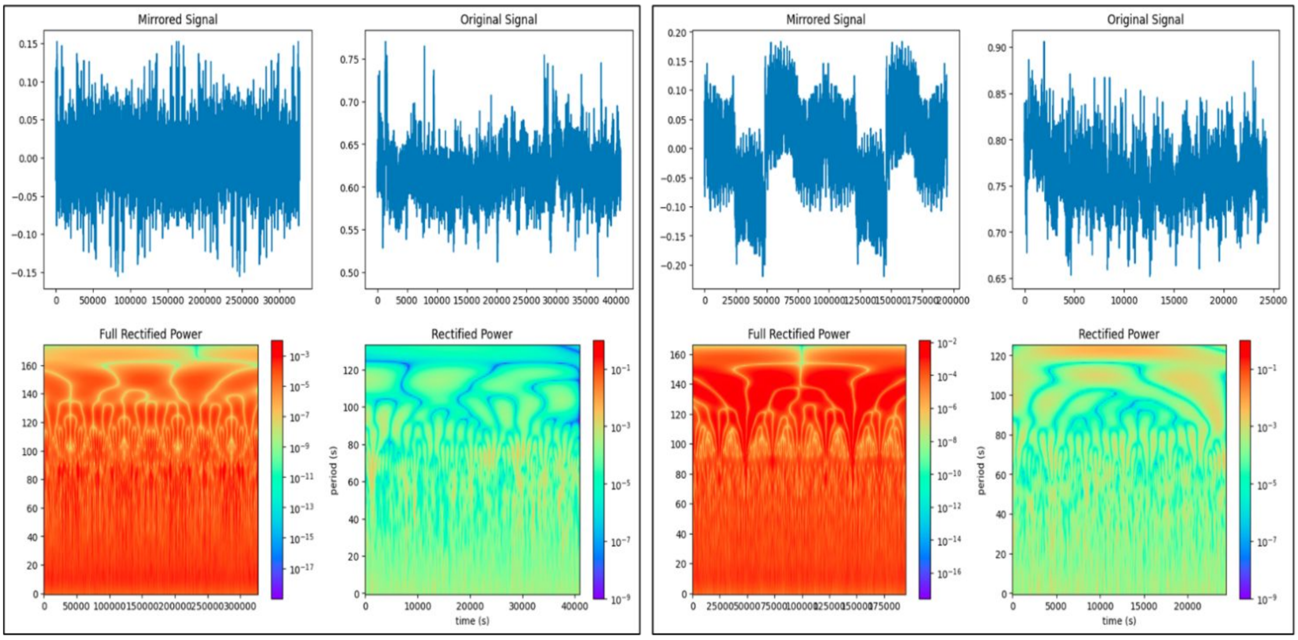

Drainage Imbibition

Figure S7: Wavelet analysis for brine fractional flow of 5% (f_w=0.05).

Reference

Spurin, C., Roberts, G. G., O'Malley, C. P. B., Kurotori, T., Krevor, S., Blunt, M. J., & Tchelepi, H. (2023). Pore-scale fluid dynamics resolved in pressure fluctuations at the Darcy scale. Geophysical Research Letters,50, e2023GL104473. https://doi.org/10.1029/2023GL104473
